# Supplementary material for: Utilizing High-Resolution Mass Spectrometry Data Mining Strategy in R Programming Language for Rapid Annotation of Absorbed Prototypes and Metabolites of Gypenosides
Source: Molecules. 2025 Feb 7;30(4):779. doi: 10.3390/molecules30040779 (PMC11858763; doi:10.3390/molecules30040779)
Supplement: Supplementary file 1 [file molecules-30-00779-s001.zip › Supplementary Materials.pdf]

Utilizing High-Resolution Mass Spectrometry Data Mining Strategy in R Programming Language for Rapid Annotation of Absorbed Prototypes and Metabolites of Gypenosides

Xiaoshan Li [1], Qianru Zhang [1], Yuqin Li [1], Lin Qin [1], Di Wu [1], Daopeng Tan [1], Jian Xie [1], Jiajia Wu [2], Qingping Yang [1], Yanliu Lu [3], Yongxia Zhao [1], Qingjie Fan [1], Xingdong Wu [1], *, Yuqi He [1], **

[1] Guizhou Engineering Research Center of Industrial Key-Technology for Dendrobium Nobile, Zunyi Medical University, 6 West Xue-Fu Road, Zunyi 563009, China; li_roseixx@163.com (X.L.); zhangqianru@zmu.edu.cn (Q.Z.); Lyq1286292@163.com (Y.L.); qinlin1115@163.com (L.Q.); wd_32677@126.com (D.W.); tandp@zmu.edu.cn (D.T.); xiejian@zmu.edu.cn (J.X.); yqp0513@163.com (Q.Y.); x.y.z.100@163.com (Y.Z.); fanqj@zmu.edu.cn (Q.F.); wuxingdong@zmu.edu.cn (X.W.); yqhe.pharm@foxmail.com (Y.H.)

[2] Shanghai Key Laboratory for Molecular Engineering of Chiral Drugs, School of Pharmacy, Shanghai Jiao Tong University, Shanghai 200240, China; wujiajia_1160@126.com (J.W.)

[3] Key Lab of the Basic Pharmacology of The Ministry of Education, Zunyi Medical University, 6 West Xue-Fu Road, Zunyi 563009, China; yanliu.lu@foxmail.com (Y.L.)

* Corresponding author: wuxingdong@zmu.edu.cn (X.W.)

** Corresponding authors: yqhe.pharm@foxmail.com (Y.H.)

Figure S1. The total ion current chromatograms (TIC) of blank serum (green) and GPs-administered serum.

Figure S2. The proportion of exogenous components detected in mice serum at different time points.

Figure S3. The total ion current chromatograms (TIC) of blank serum (red) and GPs-administered serum (green).

Figure S4. The extracting ion chromatogram (XIC) of GPs identified absorbed prototypes.

Figure S5. The time points of blood samples collection.

Table S1. Information on 72 identified chemical components of Gypenosides.

Table S2. The 36 prototypes identificated from serum administrated with gypenosides

Table S3. The peak list of screening metabolites after oxidation and deoxidized reactions of GPs.

Table S4. The peak list of screening metabolites after methylation, demethylation reactions of GPs.

Table S5. The 108 metabo lites identified from mice serum administrated with Gypenosides.

Table S6. Information on known chemical compounds in Gypenosides.

Table S7. Processes of 71 common metabolic reactions in the organism.

Table S8. Database of 71 virtual metabolites generated from 333 known chemical compounds of Gypenosides.

Supplementary Note 1. R programming code for generating virtual metabolites based on 333 known chemical compounds of Gypenosides.

Supplementary Note 2. R programming code for generating PMDF to screen potential absorption prototypes and metabolites of Gypenosides.

Supplementary Note 3. R programming code for using PMDF to screen potential absorption prototypes and metabolites of Gypenosides.

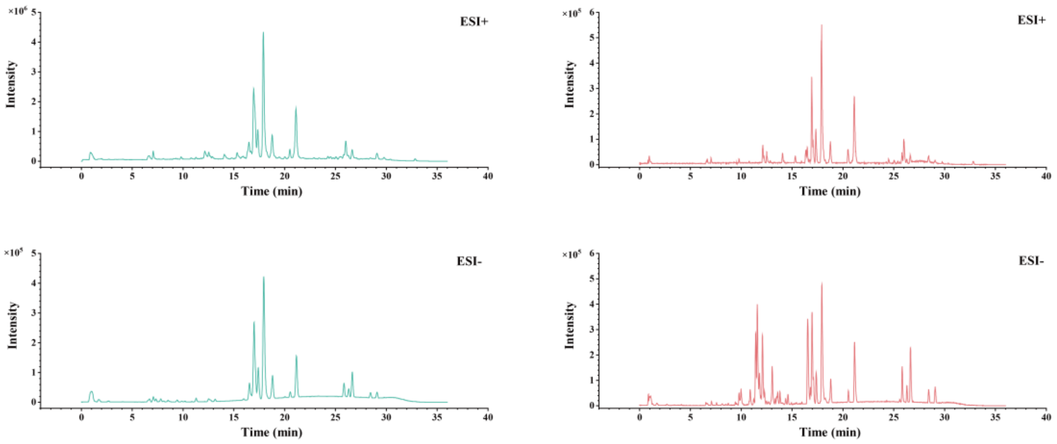

Figure S1. The total ion current chromatograms (TIC) of blank serum (green) and GPs-administered (red) serum.

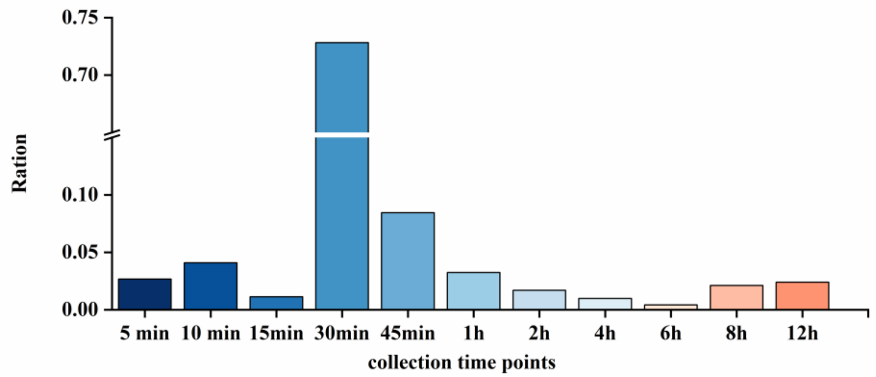

Figure S2. The proportion of exogenous components detected in mice serum at different time points.

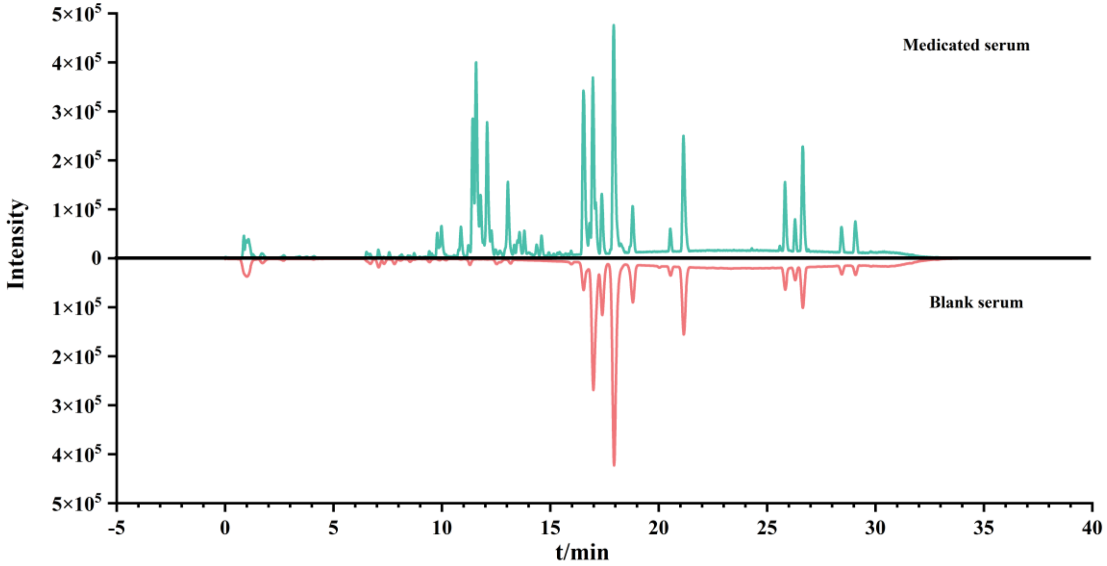

Figure S3. The total ion current chromatograms (TIC) of blank serum (red) and GPs-administered serum (green).

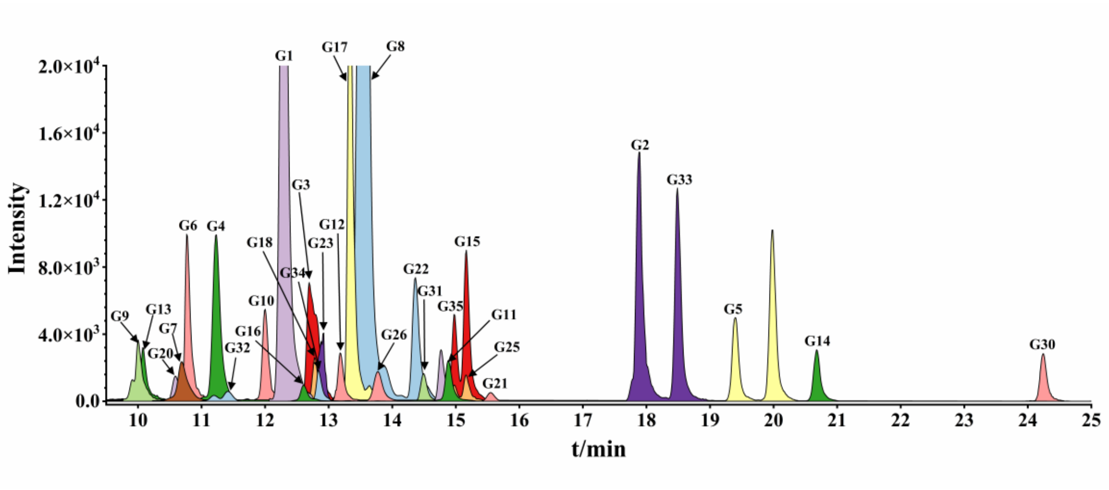

Figure S4. The extracting ion chromatogram (XIC) of GPs identified absorbed prototypes.

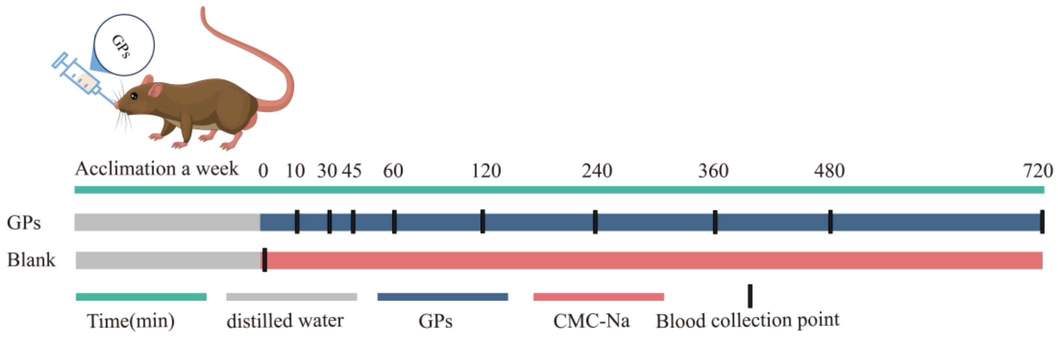

Figure S5 The time points of blood samples collection.

Table S1

NO	t/min	Calculated value(m/z)	$[M-H]^-$	$[M+FA-H]^-$	Formula	Error (ppm)	Fragment ions	Compounds
1	11.44	915.5323	915.5330	961.5388	$C_{47}H_{80}O_{17}$	2.1	915.5271, 783.4853, 637.4390, 475.3804, 161.0427	β-D-Glucopyranoside, (3β,12β,24R)-3,12,24-dihydroxydammar-25-en-20-yl O-β-D-xylopyranosyl-(1→3)-O-6-deoxy-α-L-mannopyranosyl-(1→6)- (ACI)
2	10.51	1077.5851	1077.5860	1123.5937	$C_{53}H_{90}O_{22}$	0.8	945.5447, 799.4853, 161.0461	Ginsenoside Rb2
3	12.32	883.5061	883.5046	929.5114	$C_{46}H_{76}O_{16}$	-1.7	883.4958, 751.4557, 605.4026, 473.3567, 389.2680, 149.0451, 131.0337	Gylongiposide I
4	15.13	883.5061	883.5078	929.5135	$C_{46}H_{76}O_{16}$	2	883.4993, 751.4534	Gypenoside UL3
5	13.36	899.5374	899.5371	945.5417	$C_{47}H_{80}O_{16}$	-0.3	899.5312, 767.4877, 753.4734, 621.4349, 459.3783	β-D-Glucopyranoside, (3β)-20,21-dihydroxydammar-24-en-3-yl O-6-deoxy-α-L-mannopyranosyl-(1→2)-O-[β-D-xylopyranosyl-(1→3)]-(ACI)
6	10.08	913.4802	913.4837	959.4899	$C_{46}H_{74}O_{18}$	3.8	913.4731, 781.4349, 681.3843, 667.3637, 535.3224, 403.3224, 131.0332	Dammar-24-ene-19,21-dial, 3-[(O-6-deoxy-α-L-mannopyranosyl-(1→2)-O-[β-D-xylopyranosyl-(1→3)]-α-L-arabinopyranosyl)oxy]-20,23,26-trihydroxy-, cyclic 21,23-hemiacetal, (3β,20ξ,21R,23R,24E)- (ACI)
7	10.58	913.5166	913.5198	959.5254	$C_{47}H_{78}O_{17}$	3.5	913.5081, 781.4693, 635.4100, 473.3533, 389.3050, 113.0191	Phanoside/(20S,21R,23R)-Phanoside;
8	12.81	913.5166	913.5143	959.5254	$C_{47}H_{78}O_{17}$	-2.6	913.5058, 781.4689, 635.4105, 473.3633, 389.3000, 161.0574	β-D-Glucopyranoside, (3β,20ξ)-21,23-epoxy-20,21-dihydroxydammar-24-en-3-yl O-6-deoxy-α-L-mannopyranosyl-(1→2)-O-[β-D-xylopyranosyl-(1→3)]- (9CI, ACI)
9	12.82	753.4795	753.4737	799.4846	$C_{41}H_{70}O_{12}$	-5.8	753.4737, 621.3800, 566.3674, 475.3704, 131.0310	Gypenoside XIII
10	14.52	753.4795	753.4795	799.4868	$C_{41}H_{70}O_{12}$	0.1	753.4770, 621.4229, 459.3826	Gypenoside LXXVIII

11	14.89	765.4431	765.4438	811.4494	$C_{41}H_{66}O_{13}$	1	765.4354, 633.3970, 471.3579, 359.2917, 161.0438	Gypenbioside B
12	15.66	765.4431	765.4438	811.4572	$C_{41}H_{66}O_{13}$	1	765.4369, 633.3937, 535.9687, 505.1296, 131.0326	Gypenbioside A
13	10.00	897.4853	897.4850	943.4960	$C_{46}H_{74}O_{17}$	-0.4	943.4905, 897.4796, 765.4382, 619.3840, 681.3824, 535.3299, 487.3430, 403.2828, 375.2889, 149.0451	Gypenoside UL4
14[a]	11.95	897.4853	897.4858	943.4912	$C_{46}H_{74}O_{17}$	0.5	897.5758, 765.4363, 681.3813, 535.3238, 403.2831, 131.0336	Gypenoside A
15[a]	12.82	881.4904	881.4861	927.4969	$C_{46}H_{74}O_{16}$	-4.9	881.4806, 749.4427, 603.3846, 471.3579, 389.2711, 131.0334	Dammar-24-en-21-oic acid, 3-[(O-6-deoxy-α-L-mannopyranosyl-(1→2)-O-[β-D-xylopyranosyl-(1→3)]-α-L-arabinopyranosyl)oxy]-20,23-dihydroxy-, γ-lactone, (3β,23R)- (ACI)
16	13.87	881.4904	881.4862	927.4986	$C_{46}H_{74}O_{16}$	-4.8	881.4802, 749.4410, 603.3824, 471.3471, 359.2959	Dammar-24-en-19-al, 3-[(O-6-deoxy-α-L-mannopyranosyl-(1→2)-O-[β-D-xylopyranosyl-(1→3)]-α-L-arabinopyranosyl)oxy]-21,23-epoxy-20-hydroxy-, (3β,20S,23S)- (ACI)
17	10.07	931.5272	931.5268	977.5277	$C_{47}H_{80}O_{18}$	-0.4	977.5218, 931.5196, 799.4805, 681.3811, 635.4342, 491.3709,	Gypenoside LXIV
18	16.87	751.4638	751.4630	797.4713	$C_{41}H_{68}O_{12}$	-1.1	751.4923, 605.4473, 507.0526, 361.2058	Dammar-24-en-19-al, 3-[[2-O-(6-deoxy-α-L-mannopyranosyl)-β-D-xylopyranosyl]oxy]-20,21-dihydroxy-, (3β)- (ACI)
19	13.12	751.4638	751.4652	797.4703	$C_{41}H_{68}O_{12}$	1.9	751.4623, 619.4237, 456.2543, 161.0471, 101.0268	Gypenoside XXIX
20	13.71	911.5010	911.5010	957.5060	$C_{47}H_{76}O_{17}$	0	911.4944, 779.4652, 633.3931, 567.3651, 359.2918, 161.0439, 131.0345	Dammar-24-en-21-oic acid, 3-[(O-6-deoxy-α-L-mannopyranosyl-(1→2)-O-[β-D-xylopyranosyl-(1→3)]-β-D-glucopyranosyl)oxy]-20,23-dihydroxy-, γ-lactone, (3β,20R,23R)- (9CI, ACI)

21	14.44	911.5010	911.5019	957.5079	$C_{47}H_{76}O_{17}$	-0.4	911.4849, 779.4578, 633.3905, 161.0778	Dammar-24-en-21-oic acid, 3-[(O-6-deoxy-α-L-mannopyranosyl-(1→2)-O-[β-D-xylopyranosyl-(1→3)]-β-D-glucopyranosyl)oxy]-20,23-dihydroxy-, γ-lactone, (3β,23S)- (9CI, ACI)
22[a]	10.77	1045.5589	1045.5611	1091.5619	$C_{52}H_{86}O_{21}$	2.1	913.5111, 751.4562	Gypenoside XLIX
23	11.29	1061.5902	1061.5898	1107.5955	$C_{53}H_{90}O_{21}$	0.9	929.5430, 915.5175, 783.4912, 621.4337, 459.4458	β-D-Glucopyranoside, (3β)-21-(β-D-glucopyranosyloxy)-20-hydroxydammar-24-en-3-yl O-6-deoxy-α-L-mannopyranosyl-(1→2)-O-[β-D-xylopyranosyl-(1→3)]- (9CI, ACI)
24	19.49	969.5792	969.5796	1015.5843	$C_{51}H_{86}O_{17}$	0.4	969.5669, 837.5308, 691.4734, 603.1284, 438.0794, 237.3281, 161.0434, 149.0443	Dammar-24-en-21-al, 3-[(O-6-deoxy-α-L-mannopyranosyl-(1→2)-O-[β-D-xylopyranosyl-(1→3)]-β-D-glucopyranosyl)oxy]-20,23-dihydroxy-, cyclic 21,23-(propyl acetal), (3β,20ξ,21R,23S)- (ACI)
25	20.32	939.5687	939.5685	985.5735	$C_{50}H_{84}O_{16}$	-0.2	939.5627, 807.5179, 661.4678, 459.3691, 131.0327	Dammar-24-en-21-al, 3-[(O-6-deoxy-α-L-mannopyranosyl-(1→2)-O-[β-D-xylopyranosyl-(1→3)]-α-L-arabinopyranosyl)oxy]-20,23-dihydroxy-, cyclic 21,23-(butyl acetal), (3β,20ξ,21R,23S)- (ACI)
26	9.80	927.4959	927.4974	973.5036	$C_{47}H_{76}O_{18}$	1.7	927.4903, 795.4483, 653.6297, 487.3699	Gypenoside UL5
27	10.95	927.4959	927.4915	973.5055	$C_{47}H_{76}O_{18}$	-4.7	927.4894, 795.4477, 653.8532, 544.0041, 161.0467	23β-H-3β,20ξ,21α-trihydroxy-12-oxo-21,23-epoxydammar-24-ene-3-O-[α-L-rhamnopyranosyl(1→2)][β-D-glucopyranosyl(1→3)]-α-L-arabinopyranoside
28	12.45	927.4959	927.4966	973.5029	$C_{47}H_{76}O_{18}$	0.8	927.4896, 795.4589, 765.1695, 608.4011	Dammar-24-en-21-al, 3-[(O-6-deoxy-α-L-mannopyranosyl-(1→2)-O-[β-D-glucopyranosyl-(1→3)]-α-L-arabinopyranosyl)oxy]-20,23-dihydroxy-12-oxo-, cyclic 21,23-hemiacetal, (3β,20ξ,21S,23R)- (ACI)
29	18.24	953.5479	953.5477	999.5515	$C_{50}H_{82}O_{17}$	-0.2	953.5482, 821.5076, 675.4438	Dammar-24-en-21-al, 3-[(O-6-deoxy-α-L-mannopyranosyl-(1→2)-O-[β-D-xylopyranosyl-(1→3)]-α-L-arabinopyranosyl)oxy]-20,23-dihydroxy-, cyclic 21,23-(propyl acetal), (3β,20ξ,21R,23S)- (ACI)
30	15.75	953.5115	953.5120	999.5128	$C_{49}H_{78}O_{18}$	0.5	953.5032, 821.4669, 779.4544, 635.0795, 583.7776, 471.3393	Dammar-24-en-21-oic acid, 3-[(O-6-deoxy-α-L-mannopyranosyl-(1→2)-O-[β-D-xylopyranosyl-(1→3)]-6-O-acetyl-β-D-

								glucopyranosyl)oxy]-20,23-dihydroxy-, γ-lactone, (3β,23S)- (9CI, ACI)
31	13.79	925.5166	925.5146	971.5211	$C_{48}H_{78}O_{17}$	-2.2	925.5186, 779.4562, 651.9584	Dammar-24-ene-19,21-dial, 3-[(O-6-deoxy-α-L-mannopyranosyl-(1→2)-O-[β-D-xylopyranosyl-(1→3)]-α-L-arabinopyranosyl)oxy]-20,23-dihydroxy-, cyclic 21,23-(ethyl acetal), (3β,20ξ)- (9CI, ACI)
32	10.65	1075.5695	1075.5708	1121.5792	$C_{53}H_{88}O_{22}$	1.3	913.5256, 751.4645	Gypenoside XLVIII
33	11.52	783.4900	783.4909	829.4976	$C_{42}H_{72}O_{13}$	3.5	783.4839, 697.4137, 637.4292, 475.3538, 161.0423	Gynosaponin TN 2
34	9.55	915.4959	915.4968	961.5036	$C_{46}H_{76}O_{18}$	1	915.4872, 783.4500, 637.3898, 505.3503, 451.3162	18-Norandrostan-19-al, 3-[(O-6-deoxy-α-L-mannopyranosyl-(1→2)-O-[β-D-xylopyranosyl-(1→3)]-α-L-arabinopyranosyl)oxy]-4,4,8,14-tetramethyl-17-[(1S)-1,2,4-trihydroxy-3-(1-hydroxy-1-methylethyl)cyclopentyl]-, (3β,5α,17β)- (9CI, ACI)
35	10.73	1047.5745	1047.5723	1093.5763	$C_{52}H_{88}O_{21}$	-2.1	915.5923, 753.4819	Gypenoside XV
36	12.09	769.4744	769.4846	815.4803	$C_{41}H_{70}O_{13}$	10.3	769.4689, 637.4224, 475.3750, 161.0458	Gypenoside XXI/Gypenoside LXXVII
37	12.82	929.5115	929.5114	975.4985	$C_{47}H_{78}O_{18}$	-0.2	929.5419, 767.4920, 647.3386, 615.2136, 61.9866	Gypenoside XXV
38	13.01	797.4693	797.4699	843.4656	$C_{42}H_{70}O_{14}$	0.8	797.4627, 665.4230, 549.6131	β-D-Xylopyranoside, (3β,12β,23S,24R)-23-(acetyloxy)-20,25-epoxy-12,24-dihydroxydammaran-3-yl 2-O-β-D-xylopyranosyl- (9CI)
39	10.14	1107.5957	1107.5956	1153.6075	$C_{54}H_{92}O_{23}$	-0.1	945.5367, 710.2190, 253.0479	Ginsenoside Rb1
40	10.09	931.5272	931.5197	977.5341	$C_{47}H_{80}O_{18}$	-8	931.5197, 799.4858, 491.3663	Gypenoside LVII
41	10.57	899.5010	899.5015	945.5080	$C_{46}H_{76}O_{17}$	0.6	899.4928, 767.4522, 621.3952, 489.3563	18-Norandrostan-19-al, 3-[(O-6-deoxy-α-L-mannopyranosyl-(1→2)-O-[β-D-xylopyranosyl-(1→3)]-α-L-arabinopyranosyl)oxy]-17-[1,2-dihydroxy-3-(1-hydroxy-1-methylethyl)cyclopentyl]-, (3β,5α,17β)-(9CI)

42	15.48	893.4904	893.4903	939.4960	$C_{47}H_{74}O_{16}$	-0.1	893.4797, 761.4448, 615.3824, 453.3298, 161.0489, 116.9254	Dammar-24-en-21-al, 3-[(O-6-deoxy-α-L-mannopyranosyl-(1→2)-O-[β-D-xylopyranosyl-(1→3)]-α-L-arabinopyranosyl)oxy]-20,23-dihydroxy-, cyclic 21,23-(butyl acetal), (3β,20ξ,21R,23S)-(ACI)
43[a]	11.57	945.5428	945.5431	991.5492	$C_{48}H_{82}O_{18}$	0.3	945.5388, 783.4987, 621.2630, 323.1104	Ginsenoside Rd
44	9.38	961.5378	961.5388	——	$C_{48}H_{82}O_{19}$	1.1	961.5325, 799.4643, 769.0936, 613.9710, 187.6362	Gypenoside GC6
45	13.02	737.4482	737.4472	783.4515	$C_{40}H_{65}O_{12}$	-1.3	737.4475, 605.4056, 473.3679, 391.2825, 131.0345	(3β)-20,21-Dihydroxy-3-[(3-O-β-D-xylopyranosyl-α-L-arabinopyranosyl)oxy]dammar-24-en-19-al
46	8.90	947.5221	947.5241	993.5272	$C_{47}H_{80}O_{19}$	2.1	947.5173, 815.4538, 635.5987	β-D-Glucopyranoside, (3β,12β,23S,24R)-20,25-epoxy-12,23,24-trihydroxydammaran-3-yl O-β-D-glucopyranosyl-(1→2)-O-[β-D-xylopyranosyl-(1→6)]- (9CI)
47	16.79	929.5479	929.5489	975.4172	$C_{48}H_{82}O_{17}$	1	975.4152, 929.5423, 116.9266	Gypenoside X
48	16.95	637.4321	637.4315	——	$C_{36}H_{62}O_{9}$	-1	637.4231, 475.3729, 161.0441	Gypenoside LXXVI
49	16.72	621.4372	621.4390	667.4440	$C_{36}H_{62}O_{8}$	2.9	621.4341, 459.3846, 161.0346	Ginsenoside Rh2
50	9.43	1063.5695	1063.5685	1109.5758	$C_{52}H_{88}O_{22}$	-0.9	931.5340, 752.3564	Dammaran-19-al, 3-[(O-6-deoxy-α-L-mannopyranosyl-(1→2)-O-[β-D-xylopyranosyl-(1→3)]-α-L-arabinopyranosyl)oxy]-21-(β-D-glucopyranosyloxy)-20,25-dihydroxy-, (3β)- (ACI)
51	12.15	895.4697	895.4704	941.4747	$C_{46}H_{72}O_{17}$	0.8	895.4612, 763.4244, 617.3649, 485.3273, 373.2728	Dammar-24-en-21-oic acid, 3-[(O-6-deoxy-α-L-mannopyranosyl-(1→2)-O-[β-D-xylopyranosyl-(1→3)]-α-L-arabinopyranosyl)oxy]-20,23-dihydroxy-19-oxo-, γ-lactone, (3β,23S)- (ACI)
52	13.22	941.5057	941.5078	987.5168	$C_{48}H_{78}O_{18}$	2.3	941.5006, 779.4534, 633.3934, 521.3419, 359.2920, 161.0454	Dammar-24-en-21-oic acid, 3-[(O-6-deoxy-α-L-mannopyranosyl-(1→2)-O-[β-D-glucopyranosyl-(1→3)]-β-D-glucopyranosyl)oxy]-20,23-dihydroxy-, γ-lactone, (3β,23S)- (9CI)
53	13.89	779.4587	779.4608	825.4663	$C_{42}H_{68}O_{13}$	2.7	779.4514, 633.3954, 359.2969, 161.0429	Dammar-24-en-21-oic acid, 3-[[2-O-(6-deoxy-α-L-mannopyranosyl)-β-D-glucopyranosyl]oxy]-20,23-dihydroxy-, γ-lactone, (3β,23R)-(ACI)

54	14.68	779.4587	779.4608	825.4663	$C_{42}H_{68}O_{13}$	2.7	779.4583, 697.3837, 518.8013, 443.0280, 161.0438	Dammar-24-en-21-oic acid, 3-[[2-O-(6-deoxy-α-L-mannopyranosyl)-β-D-glucopyranosyl]oxy]-20,23-dihydroxy-, γ-lactone, (3β,23S)-(ACI)
55	12.64	767.4587	767.4605	813.4627	$C_{41}H_{68}O_{13}$	1.3	767.4520, 635.4130, 473.3562, 161.0419	Gynoside E
56	10.73	813.4278	813.4205	859.4351	$C_{41}H_{65}O_{16}$	9	813.4261, 681.3848, 535.3223, 403.2825	Gypenoside GD1
57	8.79	1077.5487	1077.5470	1123.5578	$C_{52}H_{86}O_{23}$	-1.6	945.5063, 777.2900, 701.0373	Dammar-23-en-19-al, 3-[(O-6-deoxy-α-L-mannopyranosyl-(1→2)-O-[β-D-xylopyranosyl-(1→3)]-α-L-arabinopyranosyl)oxy]-21-(β-D-glucopyranosyloxy)-25-hydroperoxy-20-hydroxy-, (3β,23E)- (9CI)
58	10.43	1105.5800	1105.5796	1151.5905	$C_{54}H_{90}O_{23}$	-0.4	943.5120, 818.2893, 554.4560	Gypenoside XXXIV
59	17.47	827.4799	827.4830	873.4866	$C_{43}H_{72}O_{15}$	3.8	827.4745, 695.4328, 549.3881	β-D-Glucopyranoside, (3β,12β,23S,24R)-23-(acetyloxy)-20,25-epoxy-12,24-dihydroxydammaran-3-yl 2-O-β-D-xylopyranosyl- (9CI)
60	12.09	943.5272	943.5235	989.5276	$C_{48}H_{80}O_{18}$	-3.9	943.5206, 781.4845, 614.2080, 161.0419	Dammar-24-en-21-oic acid, 3-[(O-6-deoxy-α-L-mannopyranosyl-(1→2)-O-[β-D-glucopyranosyl-(1→3)]-β-D-glucopyranosyl)oxy]-20-hydroxy-, (3β)- (ACI)
61	13.17	785.4540	785.4508	831.4406	$C_{41}H_{70}O_{14}$	-4.1	785.4270, 653.3844, 507.3285, 149.0425	β-D-Glucopyranoside, (3β,12β,23S,24R)-20,25-epoxy-12,23,24-trihydroxydammaran-3-yl 2-O-β-D-xylopyranosyl- (9CI)
62	13.71	869.5268	869.5259	915.5331	$C_{46}H_{78}O_{15}$	-1	869.5192, 737.4806, 591.4210, 459.3831, 208.0994	α-L-Arabinopyranoside, (3β)-20,21-dihydroxydammar-24-en-3-yl *O*-6-deoxy-α-L-mannopyranosyl-(1→2)-*O*-[β-D-xylopyranosyl-(1→3)]-(ACI)
63	11.73	959.5221	959.5200	1005.5282	$C_{48}H_{80}O_{19}$	-2.2	959.5172, 797.4510, 635.3806, 117.9298	Gypenoside XXIV
64	9.61	1061.5538	1061.5577	1107.5621	$C_{52}H_{86}O_{22}$	3.7	977.4919, 929.5079, 783.4511, 651.4538, 496.6839, 161.0241, 120.6925	Dammar-25-en-19-al, 3-[(O-6-deoxy-α-L-mannopyranosyl-(1→2)-O-[β-D-xylopyranosyl-(1→3)]-α-L-arabinopyranosyl)oxy]-21-(β-D-glucopyranosyloxy)-20,24-dihydroxy-, (3β,24S)- (9CI)
65	17.80	1031.5796	1031.5784	1077.5813	$C_{52}H_{88}O_{20}$	-1.2	985.4493, 853.5297, 654.7676, 673.0040, 635.0523, 403.8786	(3β)-3-[(O-6-Deoxy-α-L-mannop yranosyl-(1→2)-O-[β-D-xylopyr anosyl-(1→3)]-α-L-arabinopyran osyl)oxy]-20-hydroxydammar-24 -en-21-yl β-D-glucopyranoside

66	25.29	473.3636	473.3624	——	$C_{30}H_{50}O_4$	-2.6	473.3573, 425.3413, 359.2913, 283.2194	Gypensapogenin P
67	13.94	955.5213	955.5186	1001.5339	$C_{49}H_{80}O_{18}$	0.7	955.5186, 823.4878, 739.4220, 551.4360, 389.3024	β-D-Glucopyranoside, (3β,20ξ)-21,23-epoxy-20,21-dihydroxydammar-24-en-3-yl *O*-6-deoxy-α-L-mannopyranosyl-(1→2)-*O*-[β-D-xylopyranosyl-(1→3)]-, 6-acetate (9CI, ACI)
68	24.37	471.3480	471.3478	——	$C_{30}H_{48}O_4$	-0.4	471.3469, 389.2696, 304.1990, 116.9284	Gypensapogenin E/F
69	14.01	943.5272	943.5249	——	$C_{48}H_{80}O_{18}$	-2.4	943.5197, 897.5176, 765.4781, 751.4631, 161.0443	Gymnemaside II
70[a]	14.75	783.4900	783.4906	829.4864	$C_{42}H_{72}O_{13}$	0.7	783.4906, 621.4375, 161.0463	Gypenoside LXXV
71[a]	13.40	799.4849	799.4843	845.4907	$C_{42}H_{72}O_{14}$	-0.8000	845.4915, 799.4843, 637.4369, 161.0451	Gypenoside LI
72	18.96	953.5479	953.5464	999.5494	$C_{50}H_{82}O_{17}$	-1.6	953.5495, 821.5030,675.4477, 131.0341	Dammar-24-en-21-al, 3-[(O-6-deoxy-α-L-mannopyranosyl-(1→2)-O-[β-D-xylopyranosyl-(1→3)]-α-L-arabinopyranosyl)oxy]-20,23-dihydroxy-, cyclic 21,23-(propyl acetal), (3β,20ξ,21R,23S)- (ACI)

a: components were identified by reference standard

Table S2

NO	t/min	Calculated value(m/z)	$[M-H]^-$	$[M+FA-H]^-$	Formula	Error (ppm)	Fragment ions	Compounds
1	12.28	883.5060	883.5053	929.5100	$C_{46}H_{76}O_{16}$	-0.9	883.5054, 751.4645, 605.4027, 473.3575	Gylongiposide I
2	17.86	953.5479	953.5451	999.5516	$C_{43}H_{68}O_{18}$	-2.8	953.5468, 821.5072, 675.4479	Dammar-24-en-21-al, 3-[(O-6-deoxy-α-L-mannopyranosyl-(1→2)-O-[β-D-xylopyranosyl-(1→3)]-α-L-arabinopyranosyl)oxy]-20,23-dihydroxy-, cyclic 21,23-(propyl acetal), (3β,20ξ,21S,23S)- (ACI)
3	12.69	913.5167	913.5143	959.5254	$C_{47}H_{78}O_{17}$	-2.6	913.5189, 781.4790, 635.4201,	β-D-Glucopyranoside, (3β,20ξ)-21,23-epoxy-20,21-

							473.3718, 389.3020, 161.0479	dihydroxydammar-24-en-3-yl O-6-deoxy-α-L-mannopyranosyl-(1→2)-O-[β-D-xylopyranosyl-(1→3)]- (9CI, ACI)
4	11.22	1061.5538	1061.5577	1107.5625	$C_{52}H_{86}O_{22}$	3.7	977.4898, 929.5560, 783.4902, 621.4348, 459.3761, 161.0422	Dammar-25-en-19-al, 3-[(O-6-deoxy-α-L-mannopyranosyl-(1→2)-O-[β-D-xylopyranosyl-(1→3)]-α-L-arabinopyranosyl)oxy]-21-(β-D-glucopyranosyloxy)-20,24-dihydroxy-, (3β,24S)- (9CI)
5	19.41	969.5792	969.5816	1015.5828	$C_{51}H_{86}O_{17}$	2.4	969.5816, 837.5332, 691.4719, 603.1284	Dammar-24-en-21-al, 3-[(O-6-deoxy-α-L-mannopyranosyl-(1→2)-O-[β-D-xylopyranosyl-(1→3)]-β-D-glucopyranosyl)oxy]-20,23-dihydroxy-, cyclic 21,23-(propyl acetal), (3β,20ξ,21R,23S)- (ACI)
6	10.77	1045.5589	1045.558	1091.5609	$C_{52}H_{86}O_{21}$	-1.1	913.5187	Gypenoside XLIX
7	12.71	927.4959	927.4942	973.5008	$C_{47}H_{76}O_{18}$	-1.8	927.4980, 530.5530	Dammar-24-en-21-al, 3-[(O-6-deoxy-α-L-mannopyranosyl-(1→2)-O-[β-D-glucopyranosyl-(1→3)]-α-L-arabinopyranosyl)oxy]-20,23-dihydroxy-12-oxo-, cyclic 21,23-hemiacetal, (3β,20ξ,21S,23R)- (ACI)
8	13.57	911.5010	911.5004	957.5040	$C_{47}H_{76}O_{17}$	-0.6	911.5050, 779.4644, 633.3969, 161.0499	Dammar-24-en-21-oic acid, 3-[(O-6-deoxy-α-L-mannopyranosyl-(1→2)-O-[β-D-xylopyranosyl-(1→3)]-β-D-glucopyranosyl)oxy]-20,23-dihydroxy-, γ-lactone, (3β,20R,23R)- (9CI, ACI)
9	10.01	897.4853	897.4814	943.4887	$C_{46}H_{74}O_{17}$	-4.4	897.4854, 765.4430, 619.4014, 487.3386, 403.2821, 131.0312	Gypenoside UL4
10	12.09	943.5272	943.5235	989.5276	$C_{48}H_{80}O_{18}$	-3.9	943.5206	Dammar-24-en-21-oic acid, 3-[(O-6-deoxy-α-L-mannopyranosyl-(1→2)-O-[β-D-glucopyranosyl-(1→3)]-β-D-glucopyranosyl)oxy]-20-hydroxy-, (3β)- (ACI)
11	14.89	779.4587	779.4608	825.4646	$C_{42}H_{68}O_{13}$	2.7	779.4627	Dammar-24-en-21-oic acid, 3-[[2-O-(6-deoxy-α-L-

								mannopyranosyl)-β-D-glucopyranosyl]oxy]-20,23-dihydroxy-, γ-lactone, (3β,23S)- (ACI)
12	13.18	941.5057	941.5078	987.5168	$C_{48}H_{78}O_{18}$	2.3	941.5006, 779.4534, 633.3934, 521.3419, 359.2920, 161.0454	Dammar-24-en-21-oic acid, 3-[(O-6-deoxy-α-L-mannopyranosyl-(1→2)-O-[β-D-glucopyranosyl-(1→3)]-β-D-glucopyranosyl)oxy]-20,23-dihydroxy-, γ-lactone, (3β,23S)-(9CI)
13	10.07	931.5272	931.5248	977.5279	$C_{47}H_{80}O_{18}$	-2.6	931.5269, 799.4818, 681.3746	Gypenoside LXIV
14	20.68	939.5687	939.5664	985.5727	$C_{50}H_{84}O_{16}$	1.5	939.5705, 807.5271, 661.4514, 621.0639, 463.0175	Dammar-24-en-21-al, 3-[(O-6-deoxy-α-L-mannopyranosyl-(1→2)-O-[β-D-xylopyranosyl-(1→3)]-α-L-arabinopyranosyl)oxy]-20,23-dihydroxy-, cyclic 21,23-(butyl acetal), (3β,20ξ,21R,23S)- (ACI)
15	15.09	883.5061	883.5038	929.5098	$C_{46}H_{76}O_{16}$	-2.6	883.5073, 751.4745	Gypenoside UL3
16	12.63	767.4587	767.4605	813.4627	$C_{41}H_{68}O_{13}$	1.3	767.4520, 635.4130, 473.3562, 161.0419	Gynoside E
17	13.33	899.5374	899.5361	945.5398	$C_{47}H_{80}O_{16}$	-1.4	899.5352, 767.5262, 621.4389, 459.3907	β-D-Glucopyranoside, (3β)-20,21-dihydroxydammar-24-en-3-yl O-6-deoxy-α-L-mannopyranosyl-(1→2)-O-[β-D-xylopyranosyl-(1→3)]- (ACI)
18	13.87	881.4904	881.4922	927.4986	$C_{46}H_{74}O_{16}$	2	881.4949, 749.4476, 603.3846, 471.3471, 359.2959	Dammar-24-en-21-al, 3-[(O-6-deoxy-α-L-mannopyranosyl-(1→2)-O-[β-D-xylopyranosyl-(1→3)]-β-D-glucopyranosyl) oxy]-20,23-dihydroxy-, cyclic 21,23-(propyl acetal), (3β,20ξ,21R,23S)-(ACI)
19	11.51	783.4900	783.4921	829.4904	$C_{42}H_{72}O_{13}$	2.7	783.4901, 621.4275, 475.3744	Gynosaponin TN 2
20	10.58	913.5166	913.5198	959.5254	$C_{47}H_{78}O_{17}$	3.5	913.5093, 781.4645, 473.3533, 389.3050	Phanoside/(20S,21R,23R)-Phanoside
21	15.54	953.5115	953.5088	999.5128	$C_{49}H_{78}O_{18}$	-2.9	953.5099, 821.4695, 779.4584, 633.4006, 471.3420	Dammar-24-en-21-oic acid, 3-[(O-6-deoxy-α-L-mannopyranosyl-(1→2)-O-[β-D-xylopyranosyl-(1→3)]-6-O-

								acetyl-β-D-glucopyranosyl)oxy]-20,23-dihydroxy-, γ-lactone, (3β,23S)- (9CI, ACI)
22	14.37	911.5010	911.4985	957.5077	$C_{47}H_{76}O_{17}$	-2.7	911.5050, 779.4644, 633.3969	Dammar-24-en-21-oic acid, 3-[(O-6-deoxy-α-L-mannopyranosyl-(1→2)-O-[β-D-xylopyranosyl-(1→3)]-β-D-glucopyranosyl)oxy]-20,23-dihydroxy-, γ-lactone, (3β,23S)- (9CI, ACI)
23	13.12	751.4638	751.4615	797.4689	$C_{41}H_{68}O_{12}$	-3.1	751.4661, 605.4142, 473.3709, 131.0354	Dammar-24-en-19-al, 3-[(2-O-β-D-glucopyranosyl-α-L-arabinopyranosyl)oxy]-20-hydroxy-, (3β)-
24	11.41	945.5428	945.5403	991.5468	$C_{48}H_{82}O_{18}$	-2.7	945.5436, 783.4746	Ginsenoside Rd
25	12.15	895.4697	895.4721	941.4743	$C_{46}H_{72}O_{17}$	2.7	895.4703, 763.4349, 582.9181	Dammar-24-en-21-oic acid, 3-[(O-6-deoxy-α-L-mannopyranosyl-(1→2)-O-[β-D-xylopyranosyl-(1→3)]-α-L-arabinopyranosyl)oxy]-20,23-dihydroxy-19-oxo-, γ-lactone, (3β,23S)- (ACI)
26	13.77	925.5166	925.5146	971.5190	$C_{48}H_{78}O_{17}$	-2.2	925.5171, 779.4677, 651.9584	Dammar-24-ene-19,21-dial, 3-[(O-6-deoxy-α-L-mannopyranosyl-(1→2)-O-[β-D-xylopyranosyl-(1→3)]-α-L-arabinopyranosyl)oxy]-20,23-dihydroxy-, cyclic 21,23-(ethyl acetal), (3β,20ξ)- (9CI, ACI)
27	14.08	943.5272	943.5254	989.5226	$C_{48}H_{80}O_{18}$	-1.9	943.5280, 897.5222, 765.4794	Gymnemaside II
28	14.54	765.4431	765.4427	811.4474	$C_{41}H_{66}O_{13}$	-0.5	765.4451, 633.4177, 471.3452, 161.0457	Gypenbioside B
29	19.49	807.4900	807.4926	853.4953	$C_{44}H_{72}O_{13}$	3.2	807.4926, 675.4484, 599.8678, 438.9691, 131.0349	unknow
30	24.23	471.3462	471.3470	——	$C_{30}H_{48}O_4$	-1	471.3489, 389.2702	Gypensapogenin E/F
31	14.49	753.4795	753.4797	799.4818	$C_{41}H_{70}O_{12}$	0.3	753.4823, 621.4345, 161.0457	Gypenoside LXXVIII
32	11.91	897.4853	897.4831	943.4897	$C_{46}H_{74}O_{17}$	-2.5	897.4857, 765.4453, 681.3892, 535.3262, 403.2852	Gypenoside A

33	18.48	953.5479	953.5498	999.5508	$C_{50}H_{82}O_{17}$	2	953.5497, 821.5107, 675.4513	Dammar-24-en-21-al, 3-[(O-6-deoxy-α-L-mannopyranosyl-(1→2)-O-[β-D-xylopyranosyl-(1→3)]-α-L-arabinopyranosyl)oxy]-20,23-dihydroxy-, cyclic 21,23-(propyl acetal), (3β,20ξ,21R,23S)- (ACI)
34	12.71	881.4904	881.4924	927.4969	$C_{46}H_{74}O_{16}$	2.3	881.4806, 749.4427, 603.3846, 471.3579, 389.2711, 131.0334	Dammar-24-en-21-oic acid, 3-[(O-6-deoxy-α-L-mannopyranosyl-(1→2)-O-[β-D-xylopyranosyl-(1→3)]-α-L-arabinopyranosyl)oxy]-20,23-dihydroxy-, γ-lactone, (3β,23R)- (ACI)
35	14.07	915.5323	915.5295	961.5353	$C_{47}H_{80}O_{17}$	-3	915.5334, 783.4895, 475.2714	Dammar-24-en-19-al, 3-[(O-6-deoxy-α-L-mannopyranosyl-(1→2)-O-[β-D-xylopyranosyl-(1→3)]-α-L-arabinopyranosyl)oxy]-21,23-epoxy-20-hydroxy-, (3β,20S,23S)- (ACI)
36	25.66	473.3636	473.3632	——	$C_{30}H_{50}O_4$	-0.4	473.3630, 425.3554, 359.2941	Gypensapogenin p

Table S3

NO	m/z	NKM	KMD	NO	m/z	NKM	KMD	NO	m/z	NKM	KMD	NO	m/z	NKM	KMD
1	285.2423	285	0.07620532	72	453.2999	453	0.206260656	143	487.2355	487	0.308553596	214	561.3271	561	0.299685255
2	324.2533	324	0.108765518	73	454.341	454	0.166323162	144	488.3464	488	0.198894041	215	561.2915	561	0.335245503
3	327.2541	327	0.111316251	74	455.3516	455	0.156851612	145	489.3593	489	0.187125059	216	561.2908	561	0.335944722
4	328.2482	328	0.118326276	75	455.3157	455	0.192711525	146	489.2377	489	0.308589279	217	562.2804	562	0.347449722
5	342.264	342	0.118176504	76	455.3157	455	0.192711525	147	491.3017	491	0.246893968	218	567.2087	567	0.424652727
6	349.2012	349	0.188722673	77	455.3154	455	0.19301119	148	491.3145	491	0.234108261	219	567.2732	567	0.360224749
7	351.2893	351	0.102954273	78	456.3187	456	0.190831488	149	491.3747	491	0.173975481	220	568.2809	568	0.35364996

8	351.2891	351	0.103154049	79	457.2963	457	0.214323089	150	493.2293	493	0.321446352	221	569.3324	569	0.303324079
9	351.2167	351	0.175473207	80	457.3311	457	0.179561947	151	495.3619	495	0.191227641	222	569.2883	569	0.347374836
10	351.2167	351	0.175473207	81	459.3482	459	0.164714268	152	497.2912	497	0.264081923	223	570.2951	570	0.341699042
11	353.2122	353	0.182201408	82	461.2911	461	0.223983736	153	499.3429	499	0.214672878	224	575.2889	575	0.353475185
12	355.2855	355	0.111216483	83	461.2912	461	0.223883847	154	499.2905	499	0.267014368	225	576.2782	576	0.365279851
13	355.3205	355	0.076255564	84	464.2223	464	0.296056752	155	499.305	499	0.252530559	226	577.2845	577	0.360103498
14	361.2748	361	0.128604214	85	465.2992	465	0.220359233	156	499.3044	499	0.253129889	227	578.2938	578	0.351930496
15	361.2222	361	0.18114548	86	465.2992	465	0.220359233	157	499.3053	499	0.252230894	228	583.2866	583	0.364705523
16	365.1958	365	0.211982454	87	465.2987	465	0.220858675	158	500.2679	500	0.290705746	229	584.293	584	0.359429282
17	365.1959	365	0.211882566	88	465.3043	465	0.215264928	159	501.3588	501	0.201023859	230	584.2758	584	0.376610076
18	368.2793	368	0.131925531	89	465.2989	465	0.220658898	160	501.321	501	0.238781651	231	584.2933	584	0.359129617
19	369.2438	369	0.168502505	90	467.3155	467	0.20631066	161	503.3721	503	0.189971936	232	584.2791	584	0.373313761
20	370.2473	370	0.166123026	91	467.3156	467	0.206210772	162	503.2665	503	0.295454022	233	584.2932	584	0.359229505
21	377.27	377	0.151264666	92	467.3166	467	0.205211889	163	503.338	503	0.22403386	234	585.2964	585	0.357149692
22	379.2117	379	0.211732794	93	468.1866	468	0.336183342	164	505.3542	505	0.210085175	235	585.2851	585	0.368437074
23	381.2274	381	0.198283551	94	468.3201	468	0.20283241	165	507.3698	507	0.196735821	236	585.2989	585	0.354652483
24	385.2388	385	0.191362733	95	469.3304	469	0.193660524	166	509.224	509	0.344606245	237	585.2836	585	0.369935399
25	387.2543	387	0.178113267	96	469.3325	469	0.191562869	167	509.2241	509	0.344506357	238	591.3012	591	0.359054731
26	387.2531	387	0.179311927	97	469.2294	469	0.294547746	168	510.2281	510	0.341627436	239	592.2911	592	0.370260066
27	389.2685	389	0.166162349	98	469.3301	469	0.193960189	169	511.2332	511	0.337649744	240	593.2969	593	0.365583156
28	389.2688	389	0.165862684	99	469.2794	469	0.244603577	170	513.2876	513	0.285543715	241	597.3012	597	0.36575441
29	391.284	391	0.152912883	100	469.2947	469	0.229320661	171	515.338	515	0.237433218	242	598.2916	598	0.376460304
30	391.2841	391	0.152812995	101	469.2797	469	0.244303912	172	515.338	515	0.237433218	243	599.2981	599	0.371084175
31	405.2636	405	0.188922689	102	469.2796	469	0.2444038	173	515.3357	515	0.23973065	244	599.2987	599	0.370484845
32	407.2792	407	0.175573335	103	471.3102	471	0.216071195	174	515.2849	515	0.290473926	245	605.4051	605	0.270903332
33	413.2701	413	0.191362853	104	471.3103	471	0.215971307	175	515.3379	515	0.237533107	246	605.2806	605	0.395264314

34	415.2857	415	0.178013498	105	471.3105	471	0.21577153	176	515.3002	515	0.27519101	247	606.2889	606	0.388090195
35	415.2856	415	0.178113387	106	471.2409	471	0.285293814	177	517.3515	517	0.226181519	248	606.4083	606	0.268823518
36	415.2805	415	0.183207692	107	471.3481	471	0.178213515	178	517.3516	517	0.226081631	249	608.3028	608	0.376438942
37	417.2633	417	0.202621713	108	471.3472	471	0.17911251	179	517.3532	517	0.224483417	250	609.3123	609	0.368066163
38	417.3012	417	0.164764032	109	471.312	471	0.214273205	180	518.3572	518	0.221604497	251	633.3981	633	0.309160685
39	418.3047	418	0.162384554	110	471.3479	471	0.178413292	181	519.3699	519	0.210035291	252	633.3988	633	0.308461467
40	418.214	418	0.252983277	111	471.3462	471	0.180111393	182	519.3672	519	0.212732276	253	635.416	635	0.293513899
41	422.2456	422	0.225885015	112	471.3455	471	0.180810612	183	519.3672	519	0.212732276	254	635.4141	635	0.295411778
42	422.1938	422	0.277627174	113	473.2911	473	0.237383094	184	521.3855	521	0.196685937	255	635.4143	635	0.295212001
43	422.2985	422	0.173044084	114	473.3611	473	0.167461257	185	521.3836	521	0.198583815	256	641.392	641	0.32418678
44	423.1972	423	0.275347584	115	473.3726	473	0.155974098	186	526.2342	526	0.353400059	257	650.4365	650	0.289785988
45	423.2741	423	0.198533451	116	473.3644	473	0.164164942	187	527.2352	527	0.353517789	258	665.3203	665	0.422605436
46	423.274	423	0.19863334	117	473.326	473	0.202522064	188	527.2672	527	0.321553521	259	667.4433	667	0.301976006
47	425.27	425	0.2048621	118	473.3259	473	0.202621952	189	528.2702	528	0.319673484	260	667.4422	667	0.303074777
48	427.2857	427	0.191412857	119	475.2366	475	0.294055465	190	529.2145	529	0.376427901	261	667.4423	667	0.302974889
49	427.2492	427	0.227872101	120	477.2862	477	0.246744076	191	529.2824	529	0.30860372	262	679.4052	679	0.353432821
50	429.2647	429	0.214622634	121	477.342	477	0.191006383	192	529.2825	529	0.308503831	263	683.4392	683	0.323937239
51	431.193	431	0.2884758	122	478.2485	478	0.285518593	193	531.3321	531	0.261192442	264	683.4389	683	0.324236904
52	433.2955	433	0.188323479	123	479.2562	479	0.278943804	194	531.331	531	0.262291213	265	683.4369	683	0.326234671
53	433.2586	433	0.225182276	124	483.3113	483	0.228371782	195	531.3329	531	0.260393335	266	684.434	684	0.330248046
54	435.2746	435	0.211433368	125	485.3273	485	0.214622874	196	533.3485	533	0.24704398	267	687.3931	687	0.374452216
55	435.274	435	0.212032698	126	485.3271	485	0.214822651	197	533.3483	533	0.247243757	268	687.3933	687	0.374252439
56	435.274	435	0.212032698	127	485.3273	485	0.214622874	198	535.365	535	0.232795631	269	695.4088	695	0.367702652
57	436.2245	436	0.262594039	128	485.3273	485	0.214622874	199	535.3649	535	0.232895519	270	695.4088	695	0.367702652
58	437.2897	437	0.198583456	129	485.3255	485	0.216420864	200	537.38	537	0.220045607	271	699.3934	699	0.387551909
59	443.2804	443	0.21457275	130	485.3254	485	0.216520753	201	537.3804	537	0.219646053	272	699.4341	699	0.346897355

60	443.2807	443	0.214273085	131	485.3254	485	0.216520753	202	537.1987	537	0.401143165	273	699.38	699	0.400936947
61	443.1912	443	0.303673148	132	485.2897	485	0.25218089	203	539.3963	539	0.205997034	274	711.3951	711	0.399253166
62	445.2955	445	0.201722838	133	485.2202	485	0.321603285	204	539.3961	539	0.206196811	275	721.4507	721	0.354881382
63	445.2953	445	0.201922614	134	485.3272	485	0.214722763	205	543.2734	543	0.333226255	276	721.451	721	0.354581717
64	445.2964	445	0.200823842	135	485.3277	485	0.214223321	206	545.3123	545	0.296602918	277	723.38	723	0.427735664
65	445.2961	445	0.201123508	136	487.341	487	0.203171398	207	545.4189	545	0.190121949	278	724.3828	724	0.426055403
66	447.2755	447	0.223933732	137	487.3429	487	0.20127352	208	547.328	547	0.283153675	279	731.3412	731	0.475425245
67	447.2738	447	0.225631833	138	487.3413	487	0.202871733	209	547.3275	547	0.283653117	280	737.4823	737	0.341182478
68	447.3116	447	0.187874041	139	487.3415	487	0.202671957	210	549.3054	549	0.307961666	281	737.4817	737	0.341781808
69	448.2789	448	0.221654141	140	487.3054	487	0.238731647	211	551.3147	551	0.300905277	282	737.4478	737	0.375643955
70	451.3219	451	0.182051995	141	487.3054	487	0.238731647	212	553.2929	553	0.324914161				
71	453.3373	453	0.168902418	142	487.3051	487	0.239031312	213	559.225	559	0.399438022				

Table S4

NO	m/z	NKM	KMD	NO	m/z	NKM	KMD	NO	m/z	NKM	KMD	NO	m/z	NKM	KMD
1	285.2423	285	-0.332989167	72	453.2999	453	-0.444020947	143	487.3413	487	-0.496243978	214	561.2908	561	-0.469255283
2	324.2533	324	-0.356392219	73	454.341	454	-0.485451952	144	489.3593	489	-0.514885576	215	562.2804	562	-0.459169913
3	327.2541	327	-0.358146286	74	455.3516	455	-0.496373259	145	489.2377	489	-0.393246914	216	567.2087	567	-0.389036804
4	328.2482	328	-0.352562347	75	455.3157	455	-0.460461845	146	491.3017	491	-0.457903137	217	567.2732	567	-0.453557311
5	342.264	342	-0.372818493	76	455.3157	455	-0.460461845	147	491.3145	491	-0.470707207	218	568.2809	568	-0.461577696
6	349.2012	349	-0.312224087	77	455.3154	455	-0.46016175	148	491.3747	491	-0.530926346	219	569.3324	569	-0.513412007
7	351.2893	351	-0.400987972	78	456.3187	456	-0.463780736	149	493.2293	493	-0.386115993	220	569.2883	569	-0.469297986
8	351.2891	351	-0.400787909	79	457.2963	457	-0.441691552	150	495.3619	495	-0.519394026	221	570.2951	570	-0.476418085

9	351.2167	351	-0.32836489	80	457.3311	457	-0.476502616	151	497.2912	497	-0.449307422	222	575.2889	575	-0.471805801
10	351.2167	351	-0.32836489	81	459.3482	459	-0.494243927	152	499.3429	499	-0.501659734	223	576.2782	576	-0.461420336
11	353.2122	353	-0.324499334	82	461.2911	461	-0.437761648	153	499.2905	499	-0.449243074	224	577.2845	577	-0.468040276
12	355.2855	355	-0.398458513	83	461.2912	461	-0.43786168	154	499.305	499	-0.463747685	225	578.2938	578	-0.477661171
13	355.3205	355	-0.433469641	84	464.2223	464	-0.369893586	155	499.3044	499	-0.463147494	226	583.2866	583	-0.472048568
14	361.2748	361	-0.389662735	85	465.2992	465	-0.447135972	156	499.3053	499	-0.46404778	227	584.293	584	-0.47876854
15	361.2222	361	-0.337046012	86	465.2992	465	-0.447135972	157	500.2679	500	-0.426953826	228	584.2758	584	-0.461563071
16	365.1958	365	-0.311909367	87	465.2987	465	-0.446635813	158	501.3588	501	-0.518200664	229	584.2933	584	-0.479068635
17	365.1959	365	-0.312009399	88	465.3043	465	-0.452237594	159	501.321	501	-0.480388646	230	584.2791	584	-0.464864121
18	368.2793	368	-0.396389727	89	465.2989	465	-0.446835877	160	503.3721	503	-0.532140767	231	584.2932	584	-0.478968604
19	369.2438	369	-0.361196378	90	467.3155	467	-0.464077029	161	503.2665	503	-0.426507193	232	585.2836	585	-0.469683489
20	370.2473	370	-0.365015428	91	467.3156	467	-0.464177061	162	503.338	503	-0.498029926	233	585.2964	585	-0.482487558
21	377.27	377	-0.389948206	92	467.3166	467	-0.465177379	163	505.3542	505	-0.514870951	234	585.2851	585	-0.471183966
22	379.2117	379	-0.332265545	93	468.1866	468	-0.335453984	164	507.3698	507	-0.531111785	235	585.2989	585	-0.484988353
23	381.2274	381	-0.348606411	94	468.3201	468	-0.468996429	165	509.224	509	-0.385901305	236	591.2835	591	-0.471491081
24	385.2388	385	-0.361281785	95	469.3304	469	-0.479617641	166	509.2241	509	-0.386001336	237	591.3012	591	-0.489196708
25	387.2543	387	-0.377422587	96	469.3325	469	-0.481718309	167	510.2281	510	-0.390320546	238	592.2911	592	-0.479411434
26	387.2531	387	-0.376222206	97	469.2294	469	-0.378585529	168	511.2332	511	-0.395740104	239	593.2969	593	-0.485531216
27	389.2685	389	-0.392262977	98	469.3301	469	-0.479317546	169	513.2876	513	-0.450793275	240	597.3012	597	-0.491104332
28	389.2688	389	-0.392563072	99	469.2794	469	-0.428601426	170	515.3357	515	-0.499544442	241	598.2916	598	-0.481819217
29	391.284	391	-0.408403779	100	469.2947	469	-0.443906291	171	515.338	515	-0.501845173	242	599.2981	599	-0.488639221
30	391.2841	391	-0.408503811	101	469.2797	469	-0.428901522	172	515.338	515	-0.501845173	243	599.2987	599	-0.489239412
31	405.2636	405	-0.392448416	102	469.2796	469	-0.42880149	173	515.2849	515	-0.448728291	244	605.4051	605	-0.597580864
32	407.2792	407	-0.40868925	103	471.3481	471	-0.497959143	174	515.3379	515	-0.501745142	245	605.2806	605	-0.473041281
33	413.2701	413	-0.401493981	104	471.3472	471	-0.497058857	175	515.3002	515	-0.464033155	246	606.2889	606	-0.481661857
34	415.2857	415	-0.417734815	105	471.312	471	-0.461847666	176	517.3515	517	-0.51598534	247	606.4083	606	-0.601099819

35	415.2856	415	-0.417634783	106	471.3479	471	-0.49775908	177	517.3516	517	-0.516085372	248	608.3028	608	-0.496202151
36	415.2805	415	-0.412533162	107	471.3455	471	-0.495358317	178	517.3532	517	-0.517685881	249	609.3123	609	-0.506023109
37	417.2633	417	-0.395963568	108	471.3102	471	-0.460047093	179	518.3572	518	-0.52200509	250	633.3981	633	-0.599480883
38	417.3012	417	-0.433875618	109	471.3103	471	-0.460147125	180	519.3672	519	-0.532326206	251	633.3988	633	-0.600181106
39	418.3047	418	-0.437694668	110	471.3105	471	-0.460347189	181	519.3672	519	-0.532326206	252	635.416	635	-0.618022449
40	418.214	418	-0.346965831	111	471.2409	471	-0.39072506	182	519.3699	519	-0.535027065	253	635.4141	635	-0.616121845
41	422.2456	422	-0.379847627	112	471.3462	471	-0.496058539	183	521.3836	521	-0.549367295	254	635.4143	635	-0.616321908
42	422.1938	422	-0.328031158	113	473.2911	473	-0.441576895	184	526.2342	526	-0.401509482	255	641.392	641	-0.595922442
43	422.2985	422	-0.432764446	114	473.3611	473	-0.511599151	185	527.2352	527	-0.402827737	256	650.4365	650	-0.643298026
44	423.1972	423	-0.331750176	115	473.3726	473	-0.523102807	186	527.2672	527	-0.434837911	257	665.3203	665	-0.531830141
45	423.2741	423	-0.408674625	116	473.3644	473	-0.5149002	187	528.2702	528	-0.438156802	258	667.4433	667	-0.655505122
46	423.274	423	-0.408574594	117	473.326	473	-0.476487991	188	529.2145	529	-0.38275703	259	667.4422	667	-0.654404772
47	425.27	425	-0.405209196	118	473.3259	473	-0.47638796	189	529.2824	529	-0.450678618	260	667.4423	667	-0.654504804
48	427.2857	427	-0.421550063	119	475.2366	475	-0.387695442	190	529.2825	529	-0.45077865	261	679.4052	679	-0.621208256
49	427.2492	427	-0.385038458	120	477.2862	477	-0.437947087	191	531.331	531	-0.499929945	262	683.4389	683	-0.65619072
50	429.2647	429	-0.401179261	121	477.342	477	-0.493764828	192	531.3321	531	-0.501030294	263	683.4369	683	-0.654190084
51	431.193	431	-0.330092339	122	478.2485	478	-0.400553038	193	531.3329	531	-0.501830549	264	683.4392	683	-0.656490815
52	433.2955	433	-0.433260802	123	479.2562	479	-0.408573423	194	533.3485	533	-0.518071383	265	684.434	684	-0.651607099
53	433.2586	433	-0.39634907	124	483.3113	483	-0.464962691	195	533.3483	533	-0.51787132	266	687.3931	687	-0.611647908
54	435.2746	435	-0.412990032	125	485.3273	485	-0.481603652	196	535.365	535	-0.535212504	267	687.3933	687	-0.611847971
55	435.274	435	-0.412389841	126	485.3271	485	-0.481403589	197	535.3649	535	-0.535112472	268	695.4088	695	-0.629896398
56	435.274	435	-0.412389841	127	485.3272	485	-0.481503621	198	537.38	537	-0.550853147	269	695.4088	695	-0.629896398
57	436.2245	436	-0.363192041	128	485.3277	485	-0.48200378	199	537.3804	537	-0.551253275	270	699.3934	699	-0.615763251
58	437.2897	437	-0.428730707	129	485.3273	485	-0.481603652	200	537.1987	537	-0.369495505	271	699.38	699	-0.60235899
59	443.2804	443	-0.421335374	130	485.3273	485	-0.481603652	201	539.3963	539	-0.567794204	272	699.4341	699	-0.656476191
60	443.2807	443	-0.42163547	131	485.3255	485	-0.47980308	202	539.3961	539	-0.567594141	273	711.3951	711	-0.621279039

61	443.1912	443	-0.332107014	132	485.2897	485	-0.443991698	203	543.2734	543	-0.446126879	274	721.4507	721	-0.680076089
62	445.2955	445	-0.43707605	133	485.2202	485	-0.374469601	204	545.3123	545	-0.485675121	275	721.451	721	-0.680376184
63	445.2953	445	-0.436875986	134	485.3254	485	-0.479703048	205	545.4189	545	-0.592309014	276	723.38	723	-0.609989486
64	445.2964	445	-0.437976336	135	485.3254	485	-0.479703048	206	547.328	547	-0.502015988	277	724.3828	724	-0.613108313
65	445.2961	445	-0.437676241	136	487.341	487	-0.495943883	207	547.3275	547	-0.501515829	278	731.3412	731	-0.573720648
66	447.2755	447	-0.417705566	137	487.3429	487	-0.497844487	208	549.3054	549	-0.480044677	279	737.4823	737	-0.716773133
67	447.2738	447	-0.416005025	138	487.3054	487	-0.460332564	209	551.3147	551	-0.489983508	280	737.4817	737	-0.716172942
68	447.3116	447	-0.453817043	139	487.3054	487	-0.460332564	210	553.2929	553	-0.468812452	281	737.4478	737	-0.682262164
69	448.2789	448	-0.421424584	140	487.3051	487	-0.460032469	211	559.225	559	-0.402798488				
70	451.3219	451	-0.465392067	141	487.2355	487	-0.39041034	212	561.3271	561	-0.505566824				
71	453.3373	453	-0.481432838	142	487.3415	487	-0.496444042	213	561.2915	561	-0.469955505				

Table S5

NO	m/z	t/min	Parent compound	Parent compound formula	Metabolism formula	Description	Classifier	Metabolite formula	Error (ppm)
M1	349.2012	8.98	Secolongipegenin S3	$C_{24}H_{38}O_5$	$-C_4H_8$	Tert-butyl to dealkylation	Phase1	$C_{20}H_{30}O_5$	0.7
M2	415.2805	17.27	Gypensapogenin E	$C_{30}H_{48}O_4$	$-C_4H_8$	Tert-butyl to dealkylation	phase1	$C_{26}H_{40}O_4$	-9.1
M3	365.1959	8.14	Secolongipegenin S3	$C_{24}H_{38}O_5$	$-C_4H_8 + O$	Tert-butyl to alcohol1	phase1	$C_{20}H_{30}O_6$	0.1
M4	891.4522	11.75	Gypenoside LXIV	$C_{47}H_{80}O_{18}$	$-C_4H_8 + O$	Tert-butyl to alcohol1	Phase1	$C_{43}H_{72}O_{19}$	-7
M5	461.2912	13.47	Gypensapogenin P	$C_{30}H_{50}O_4$	$-C_3H_8 + O_2$	Tert-butyl to acid	phase1	$C_{27}H_{42}O_6$	3.1
M6	389.2688	14.94	Secolongipegenin S3	$C_{24}H_{38}O_5$	-O	Sulfoxide to Thioether	phase1	$C_{24}H_{38}O_4$	0.4
M7	485.2202	11.22	Secolongipegenin S3	$C_{24}H_{38}O_5$	$+SO_3$	Sulfate conjugation	Phase2	$C_{24}H_{38}SO_4$	-0.3
M8	407.2792	10.68	Secolongipegenin S3	$C_{24}H_{38}O_5$	$+H_2$	Reduction	Phase1	$C_{24}H_{40}O_5$	-2.7

M9	883.5049	15.43	21-norgypenoside B	$C_{46}H_{74}O_{16}$	$+H_2$	Reduction	phase1	$C_{46}H_{76}O_{16}$	-1.3
M10	1015.5809	21.89	Gypenoside CP2	$C_{52}H_{86}O_{19}$	$+H_2$	Reduction	phase1	$C_{52}H_{88}O_{19}$	-3.8
M11	425.2700	15.66	Gypensapogenin D	$C_{30}H_{46}O_3$	$-C_3H_8 + O$	Propyl ether to acid	phase1	$C_{27}H_{38}O_4$	0.6
M12	443.2807	13.47	Gypensapogenin F	$C_{30}H_{48}O_4$	$-C_3H_8 + O$	Propyl ether to acid	phase1	$C_{27}H_{40}O_5$	0.9
M13	485.2897	12.25	Gypensapogenin G	$C_{32}H_{50}O_5$	$-C_3H_8 + O$	Propyl ether to acid	phase1	$C_{29}H_{42}O_6$	-2.4
M14	899.5002	11.22	Gylongiposide I	$C_{46}H_{76}O_{16}$	$+O_2$-O	Oxidation+reduction	phase1	$C_{46}H_{76}O_{17}$	-0.9
M15	915.5015	13.95	Gylongiposide I	$C_{46}H_{76}O_{16}$	$+O_2$	Oxidation	phase1	$C_{46}H_{76}O_{18}$	6.1
M16	1017.5923	19.98	Gypenoside XV	$C_{52}H_{88}O_{21}$	$-O_2 + H_2$	Nitro reduction	Phase1	$C_{52}H_{90}O_{19}$	-7.9
M17	985.5780	19.14	Gypenoside XCV	$C_{50}H_{84}O_{18}$	$+CH_2$	Methylation	Phase2	$C_{51}H_{86}O_{18}$	3.9
M18	897.5203	16.93	Gylongiposide I	$C_{46}H_{76}O_{16}$	$+CH_2$	Methylation	phase2	$C_{47}H_{78}O_{16}$	-1.6
M19	461.2911	16.95	Gypsapogenin A	$C_{30}H_{48}O_5$	$-C_3H_6 + O$	Isopropyl to alcohol	phase1	$C_{27}H_{42}O_6$	0.5
M20	727.4325	10.19	Gypenoside XIII	$C_{41}H_{70}O_{12}$	$-C_3H_6 + O$	Isopropyl to alcohol	phase1	$C_{38}H_{64}O_{13}$	7.0
M21	379.2117	8.73	Secolongipegenin S3	$C_{24}H_{38}O_5$	$-C_3H_6 + O$	Isopropyl to alcohol	phase1	$C_{21}H_{32}O_6$	-2.4
M22	477.2862	12.89	Secolongipegenin S1	$C_{30}H_{48}O_6$	$-C_3H_6 + O$	Isopropyl to alcohol	phase1	$C_{27}H_{42}O_7$	-3.9
M23	817.4241	10.35	Gycomoside I	$C_{42}H_{72}O_{15}$	$-C_2H_6+O_2$	Isopropyl to acid	phase1	$C_{40}H_{66}O_{17}$	1.7
M24	515.3002	12.98	Gypensapogenin G	$C_{32}H_{50}O_5$	$-C_2H_6+O_2$	Isopropyl to acid	phase1	$C_{30}H_{44}O_7$	-2.4
M25	737.4821	14.86	Gypenoside XIV	$C_{42}H_{72}O_{12}$	$-CH_2O$	Hydroxymethylene loss	phase1	$C_{41}H_{70}O_{11}$	-3.3
M26	391.2841	13.81	3β-hydroxyetio-17β-dammaranic acid	$C_{23}H_{38}O_3$	$+O+CH_2$	Hydroxylation + methylation	phase2	$C_{24}H_{40}O_4$	-3.3
M27	435.2746	16.82	Secolongipegenin S3	$C_{24}H_{38}O_5$	$+O+CH_2$	Hydroxylation + methylation	phase2	$C_{25}H_{40}O_6$	-1.4
M28	813.5013	16.68	Gypenoside LXXV	$C_{42}H_{72}O_{13}$	$+O+CH_2$	Hydroxylation + methylation	phase2	$C_{43}H_{74}O_{14}$	0.9

M29	1107.5946	10.82	Ginsenoside Rb3	$C_{53}H_{90}O_{22}$	$+O+CH_2$	Hydroxylation + methylation	phase2	$C_{54}H_{92}O_{23}$	-1.0
M30	969.5767	20.43	(3S*,20S*,21R*,23R*)-21-O-n-butyl-3,20,21-trihydroxy-21,23-epoxydammar-24-ene 3-O-[α-ʟ-rhamnopyranosyl-(1→2)]-[β-ᴅ-xylopyranosyl-(1→3)]-α-ʟ-arabinopyranoside	$C_{50}H_{84}O_{16}$	$+O+CH_2$	Hydroxylation + methylation	phase2	$C_{51}H_{86}O_{17}$	-2.6
M31	943.5261	14.39	Phanoside	$C_{47}H_{78}O_{17}$	$+O+CH_2$	Hydroxylation + methylation	phase2	$C_{48}H_{80}O_{18}$	-1.2
M32	853.4934	20.56	Damulin C	$C_{44}H_{72}O_{14}$	$+O+CH_2$	Hydroxylation + methylation	phase2	$C_{45}H_{74}O_{15}$	-2.5
M33	913.5142	15.11	Gylongiposide I	$C_{46}H_{76}O_{16}$	$+O+CH_2$	Hydroxylation + methylation	phase2	$C_{47}H_{78}O_{17}$	-2.7
M34	1119.5588	11.58	Longipenoside GL3	$C_{48}H_{80}O_{17}$	$+C_6H_8O_7$	Hydroxylation + glucuronide conjugation	phase2	$C_{54}H_{88}O_{24}$	-0.4
M35	873.4844	17.86	Gypensapogenin T	$C_{38}H_{66}O_{10}$	$+C_6H_8O_7$	Hydroxylation + glucuronide conjugation	phase2	$C_{44}H_{74}O_{17}$	-1.1
M36	553.2981	12.72	3β-hydroxyetio-17β-dammaranic acid	$C_{23}H_{38}O_3$	$+C_6H_8O_7$	Hydroxylation + glucuronide conjugation	phase2	$C_{29}H_{46}O_{10}$	-6.7
M37	895.4739	11.32	21-norgypenoside B	$C_{46}H_{74}O_{16}$	$+O-H_2$	Hydroxylation + desaturation	phase1	$C_{46}H_{72}O_{17}$	4.7
M38	925.4802	13.40	21-norgypenoside A	$C_{47}H_{76}O_{17}$	$+O-H_2$	Hydroxylation + desaturation	phase1	$C_{47}H_{74}O_{18}$	1.1
M39	377.2700	13.40	3β-hydroxyetio-17β-dammaranic acid	$C_{23}H_{38}O_3$	+O	Hydroxylation	phase1	$C_{23}H_{38}O_4$	0.7
M40	799.4850	14.84	Gypenoside XII	$C_{42}H_{72}O_{13}$	+O	Hydroxylation	phase1	$C_{42}H_{72}O_{14}$	0.1
M41	843.4446	11.77	Longipenoside ND1	$C_{42}H_{68}O_{16}$	+O	Hydroxylation	Phase1	$C_{42}H_{68}O_{17}$	7.4
M42	931.5253	12.28	Gypenoside IX	$C_{47}H_{80}O_{17}$	+O	Hydroxylation	phase1	$C_{47}H_{80}O_{18}$	-2.0

M43	899.5002	11.22	Gylongiposide I	$C_{46}H_{76}O_{16}$	+O	Hydroxylation	phase1	$C_{46}H_{76}O_{17}$	-0.9
M44	423.2741	9.68	Secolongipegenin S3	$C_{24}H_{38}O_5$	$+H_2O$	Hydration	phase1	$C_{24}H_{40}O_6$	-2.6
M45	1110.5776	9.57	Longipenoside GL2	$C_{54}H_{86}O_{20}$	$+C_2H_3NO$	Glycine conjugation	phase2	$C_{56}H_{89}NO_{21}$	-7.1
M46	811.4847	17.49	Gypensapogenin S	$C_{37}H_{64}O_8$	$+C_6H_8O_6$	Glucuronide conjugation	phase2	$C_{43}H_{72}O_{14}$	-0.3
M47	959.5214	12.06	Gypenoside LXXV	$C_{42}H_{72}O_{13}$	$+C_6H_8O_6$	Glucuronide conjugation	phase2	$C_{48}H_{80}O_{19}$	-0.7
M48	795.4532	17.78	Gypensapogenin I	$C_{36}H_{60}O_8$	$+C_6H_8O_6$	Glucuronide conjugation	phase2	$C_{42}H_{68}O_{14}$	-0.5
M49	941.5098	16.26	Ginsenoside Rg5	$C_{42}H_{70}O_{12}$	$+C_6H_8O_6$	Glucuronide conjugation	phase2	$C_{48}H_{78}O_{18}$	-1.8
M50	469.2947	16.29	Gypensapogenin D	$C_{30}H_{46}O_3$	$-CH_4+O_2$	Ethyl to carboxylic acid	phase1	$C_{29}H_{42}O_5$	-2.7
M51	847.4336	11.00	Gypenoside GD3	$C_{42}H_{72}O_{16}$	$-CH_4+O_2$	Ethyl to carboxylic acid	phase1	$C_{41}H_{68}O_{18}$	0.4
M52	499.3044	13.74	Gypensapogenin G	$C_{32}H_{50}O_5$	$-C_2H_6$ + O	Ethyl Ether to acid	phase1	$C_{30}H_{44}O_6$	-4.2
M53	917.5071	12.85	Gylongiposide I	$C_{46}H_{76}O_{16}$	$+O+H_2O$	Epoxidation + hydrolysis	phase1	$C_{46}H_{78}O_{18}$	-4.8
M54	899.5071	13.65	Gylongiposide I	$C_{46}H_{76}O_{16}$	+O	Epoxidation	phase1	$C_{46}H_{76}O_{17}$	6.8
M55	451.3219	23.85	Gypensapogenin D	$C_{30}H_{46}O_3$	$-H_2$	Desaturation	phase1	$C_{30}H_{44}O_3$	0.3
M56	881.4896	16.86	Gylongiposide I	$C_{46}H_{76}O_{16}$	$-H_2$	Desaturation	phase1	$C_{46}H_{74}O_{16}$	-0.9
M57	473.3259	14.58	Gypensapogenin E	$C_{30}H_{48}O_4$	$-CH_2+O$	Demethylation + hydroxylation	phase1	$C_{29}H_{46}O_5$	-2.9
M58	455.3154	17.36	Gypensapogenin D	$C_{30}H_{46}O_3$	$-CH_2+O$	Demethylation + hydroxylation	phase1	$C_{29}H_{44}O_4$	-2.8
M59	473.3260	19.13	Gypensapogenin E	$C_{30}H_{48}O_4$	$-CH_2+O$	Demethylation + hydroxylation	phase1	$C_{29}H_{46}O_5$	-2.6
M60	471.3105	13.83	Gypensapogenin D	$C_{30}H_{46}O_3$	$-CH_2+O_2$	Demethylation + 2 hydroxylation	phase1	$C_{29}H_{44}O_5$	-2.3
M61	531.3329	20.70	Gypensapogenin G	$C_{32}H_{50}O_5$	$-CH_2+O_2$	Demethylation + 2 hydroxylation	phase1	$C_{31}H_{48}O_7$	0.3
M62	803.4436	10.56	Gypenoside LIX	$C_{41}H_{70}O_{14}$	$-CH_2+O_2$	Demethylation + 2 hydroxylation	Phase1	$C_{40}H_{68}O_{16}$	0.2
M63	499.3429	18.74	Gypensapogenin G	$C_{32}H_{50}O_5$	$-CH_2$	Demethylation	phase1	$C_{31}H_{48}O_5$	0

M64	899.5025	9.82	Phanoside	$C_{47}H_{78}O_{17}$	$-CH_2$	Demethylation	Phase1	$C_{46}H_{76}O_{17}$	1.7
M65	813.4334	10.86	Longipenoside ND1	$C_{42}H_{68}O_{16}$	$-CH_2$	Demethylation	Phase1	$C_{41}H_{66}O_{16}$	6.9
M66	457.3311	20.80	Gypensapogenin E	$C_{30}H_{48}O_4$	$-CH_2$	Demethylation	Phase1	$C_{29}H_{46}O_4$	-2.7
M67	801.4572	17.79	Gynosaponin TR1	$C_{36}H_{62}O_{10}$	$+C_5H_8O_5$	Decarboxylation + glucuronidation	phase2	$C_{41}H_{70}O_{15}$	-8.7
M68	929.5106	15.44	Damulin A	$C_{42}H_{70}O_{13}$	$+C_5H_8O_5$	Decarboxylation + glucuronidation	phase2	$C_{47}H_{78}O_{18}$	-1.0
M69	553.2989	11.22	Secolongipegenin S3	$C_{24}H_{38}O_5$	$+C_5H_8O_5$	Decarboxylation + glucuronidation	phase2	$C_{29}H_{46}O_{10}$	-5.3
M70	1094.5794	10.91	Gypenoside GD4	$C_{54}H_{92}O_{25}$	-COOH	Decarboxylation	phase1	$C_{53}H_{91}O_{23}$	-7.7
M71	1028.5857	19.75	Longipenoside GL4	$C_{54}H_{90}O_{21}$	-COOH	Decarboxylation	phase1	$C_{53}H_{89}O_{19}$	-6.6
M72	408.3410	26.51	Gypensapogenin D	$C_{30}H_{46}O_3$	-COOH	Decarboxylation	phase1	$C_{29}H_{45}O$	3.0
M73	931.5311	10.26	Gypenoside XXIV	$C_{48}H_{80}O_{19}$	-CO	Decarbonylation	phase1	$C_{47}H_{80}O_{18}$	4.2
M74	387.2543	14.16	Secolongipegenin S3	$C_{24}H_{38}O_5$	$-H_2O$	Alcohols dehydration	phase1	$C_{24}H_{36}O_4$	0.6
M75	953.5467	18.27	Gypenoside XCV	$C_{50}H_{84}O_{18}$	$-H_2O$	Alcohols dehydration	phase1	$C_{50}H_{82}O_{17}$	-1.3
M76	953.5503	18.33	Gypenoside XCIII	$C_{50}H_{84}O_{18}$	$-H_2O$	Alcohols dehydration	phase1	$C_{50}H_{82}O_{17}$	2.5
M77	911.4992	19.70	Gylongiposide I	$C_{46}H_{76}O_{16}$	$-H_2O$	Alcohols dehydration	Phase1	$C_{46}H_{74}O_{15}$	4.3
M78	1001.5343	14.18	Gypenoside XXIV	$C_{48}H_{80}O_{19}$	$+C_2H_2O$	Acetylation	phase2	$C_{50}H_{82}O_{20}$	1.6
M79	969.5413	18.64	Longipenoside GL3	$C_{48}H_{80}O_{17}$	$+C_2H_2O$	Acetylation	phase2	$C_{50}H_{82}O_{18}$	-1.6
M80	923.4967	10.78	21-norgypenoside B	$C_{46}H_{74}O_{16}$	$+C_2H_2O$	Acetylation	Phase2	$C_{48}H_{76}O_{17}$	-4.6

M81	501.3210	12.98	Gypensapogenin D	$C_{30}H_{46}O_3$	$+O_3$	3 Hydroxylation	phase1	$C_{30}H_{46}O_6$	-2.3
M82	683.4369	12.97	Gypensapogenin S	$C_{37}H_{64}O_8$	$+O_3$	3 Hydroxylation	phase1	$C_{37}H_{64}O_{11}$	-1.0
M83	471.3102	13.18	Gypensapogenin E	$C_{30}H_{48}O_4$	$-CH_4 + O$	2-ethoxyl to acid	phase1	$C_{29}H_{44}O_5$	-3.0
M84	453.2999	19.68	Gypensapogenin D	$C_{30}H_{46}O_3$	$-CH_4 + O$	2-ethoxyl to acid	Phase1	$C_{29}H_{42}O_4$	-2.5
M85	465.2987	24.82	Gypensapogenin A	$C_{30}H_{42}O_2$	$+O_2$	2 Hydroxylation	phase1	$C_{30}H_{42}O_4$	-5.0
M86	465.2992	25.95	Gypensapogenin B	$C_{30}H_{42}O_2$	$+O_2$	2 Hydroxylation	phase1	$C_{30}H_{42}O_4$	-3.9
M87	485.3277	13.92	Gypensapogenin D	$C_{30}H_{46}O_3$	$+O_2$	2 Hydroxylation	phase1	$C_{30}H_{46}O_5$	0.9
M88	483.3113	19.13	Dammara-24,20-dien-26-oic acid, 22-hydroxy-3-oxo-, δ-lactone, (22S)- (ACI)	$C_{30}H_{44}O_3$	$+O_2$	2 Hydroxylation	phase1	$C_{30}H_{44}O_5$	-0.6
M89	433.2955	14.90	Gypensapogenin P	$C_{30}H_{50}O_4$	$-C_4H_8 + O$	Tert-butyl to alcohol1	phase1	$C_{26}H_{42}O_5$	-1.0
M90	493.2793	9.68	Secolongipegenin S2	$C_{30}H_{50}O_7$	$-C_3H_8 + O$	Tert-butyl to acid	phase1	$C_{27}H_{42}O_8$	-2.8
M91	509.2800	8.89	Secolongipegenin S2	$C_{30}H_{50}O_7$	$-C_3H_8 + O_2$	Tert-butyl to acid + Oxidation	phase1	$C_{27}H_{42}O_9$	8.6
M92	485.3254	25.08	Gypensapogenin D	$C_{30}H_{46}O_3$	$+O_2$	2 Hydroxylation	phase1	$C_{30}H_{46}O_5$	-3.8
M93	487.3413	20.10	Gypensapogenin F	$C_{30}H_{48}O_4$	+O	Oxidation	phase1	$C_{30}H_{48}O_5$	-3.3
M94	515.3357	25.80	Gypensapogenin G	$C_{32}H_{50}O_5$	$-CH_2+O$	Demethylation + hydroxylation	phase1	$C_{31}H_{48}O_6$	-4.1
M95	519.3699	20.43	Secolongipegenin S2	$C_{30}H_{50}O_7$	$+CH_2-O$	Methylation+ Reduction	phase2	$C_{31}H_{52}O_6$	1.5
M96	535.3649	17.30	Secolongipegenin S2	$C_{30}H_{50}O_7$	$+CH_2$	Methylation	phase2	$C_{31}H_{52}O_7$	1.6
M97	683.4389	16.16	Gypensapogenin T	$C_{38}H_{66}O_{10}$	$-CH_2+O$	Demethylation + hydroxylation	phase1	$C_{37}H_{64}O_{11}$	1.9
M98	699.4341	11.32	Gypensapogenin T	$C_{38}H_{66}O_{10}$	$-CH_2+O_2$	Demethylation + 2 hydroxylation	phase1	$C_{37}H_{64}O_{12}$	2.3
M99	447.3116	15.34	Gypensapogenin P	$C_{30}H_{50}O_4$	$-C_3H_6+ O$	Tert-butyl to alcohol + Methylation	phase1	$C_{27}H_{44}O_5$	0
M100	429.2647	16.65	Gypensapogenin F	$C_{30}H_{48}O_4$	$-C_4H_{10} + O$	Propyl ether to acid + Demethylation	phase1	$C_{26}H_{38}O_5$	0.1
M101	457.2963	17.28	Gypensapogenin F	$C_{30}H_{48}O_4$	$-C_2H_6 + O$	Propyl ether to acid + methylation	phase1	$C_{28}H_{42}O_5$	0.8
M102	491.3017	18.60	Secolongipegenin S1	$C_{30}H_{48}O_6$	$-C_2H_4 + O$	Isopropyl to alcohol + methylation	phase1	$C_{28}H_{44}O_7$	0.5
M103	585.2751	9.79	Secolongipegenin S1	C30H48O6	$+SO_4-CH_2$	Hydroxylation + sulfation +	phase2	$C_{29}H_{46}O_{10}S$	2.1

						demethylation			
M104	599.2887	10.91	Secolongipegenin S1	$C_{30}H_{48}O_6$	$+SO_4$	Hydroxylation + sulfation	phase2	$C_{30}H_{48}O_{10}S$	-1.4
M105	781.4788	13.12	Gypenoside LXXV	$C_{42}H_{72}O_{13}$	$-H_2$	Desaturation	phase1	$C_{42}H_{70}O_{13}$	5.7
M106	635.4141	23.85	Compound K	$C_{36}H_{62}O_8$	$-C_6H_{10}O_5+C_6H_8O_6$	Hydrolysis + glucuronidation	phase2	$C_{36}H_{60}O_9$	-3.7
M107	377.3027	13.40	Compound K	$C_{36}H_{62}O_8$	$-C_6H_{10}O_5-C_6H_{12}$	hydrolysis	phase1	$C_{24}H_{42}O_3$	-9.1
M108	815.4812	11.58	Gypenoside LXXV	$C_{42}H_{72}O_{13}$	+2O	2 hydroxylation	phase1	$C_{42}H_{72}O_{15}$	1.7

Table S6

-

Table S7

NO	Mass	Formula	Description	Phase
1	0.9840	-NH+O	Oxidative Deamination to alcohol	1
2	1.9429	$-C_2H_6+O_2$	Isopropyl to acid	1
3	1.9793	$-CH_2+O$	Demethylation + hydroxylation	1
4	2.0157	$+H_2$	Reduction	1
5	13.9793	$+O-H_2$	Hydroxylation + desaturation	1
6	14.0157	$+CH_2$	Methylation	2
7	15.9585	$-CH_4+O_2$	Ethyl to carboxylic acid	1
8	15.9949	+O	Hydroxylation	1
9	17.9742	$-CH_2+O_2$	Demethylation + 2 hydroxylation	1
10	18.0106	$+H_2O$	Hydration	1
11	29.9742	$-H_2+O_2$	Quinone formation	1
12	30.0106	$+O+CH_2$	Hydroxylation + methylation	2
13	31.9898	$+O_2$	2 Hydroxylation	1
14	34.0055	$+H_2O_2$	Alkenes to dihydrodiol	1
15	42.0106	$+C_2H_2O$	Acelylation	2
16	47.9847	$+O_3$	3 Hydroxylation	1
17	57.0215	$+C_2H_3NO$	Glycine conjugation	2
18	79.9568	$+SO_3$	Sulfate conjugation	2
19	95.9517	$+SO_4$	Hydroxylation + sulfation	2
20	103.0092	$+C_3H_5NOS$	Cysteine conjugation	2
21	107.0041	$+C_2H_5NO_2S$	Taurine conjugation	2
22	119.0041	$+C_3H_5NO_2S$	S-Cysteine conjugation	2
23	148.0372	$+C_5H_8O_5$	Decarboxylation + glucuronidation	2
24	161.0147	$+C_5H_7NO_3S$	N-acetylcysteine conjugation	2
25	176.0321	$+C_6H_8O_6$	Glucuronide conjugation	2
26	191.9035	$+S_2O_8$	2 O-sulfate conjugation	2
27	192.0270	$+C_6H_8O_7$	Hydroxylation + glucuronide conjugation	2
28	289.0732	$+C_{10}H_{15}N_3O_5S$	Glutathione conjugation	2
29	303.0525	$+C_{10}H_{13}N_3O_6S$	Desaturation + S-Glutathione conjugation	2
30	305.0682	$+C_{10}H_{15}N_3O_6S$	S-Glutathione conjugation	2
31	321.0631	$+C_{10}H_{15}N_3O_7S$	OH + S-Glutathione conjugation	2
32	352.0642	$+C_{12}H_{16}O_{12}$	2 Glucuronide conjugation	2
33	0.0364	$-CH_4 + O$	2-ethoxyl to acid	1
34	1.0316	$-NH_3 + O$	Oxidative Deamination to ketone	1

35	1.9957	-F + OH	Oxidative Defluorination	1
36	2.0157	$-H_2$	Desaturation	1
37	4.0313	$-H_4$	Twto sequential desaturations	1
38	12.0364	$-C_2H_4 + O$	Ethyl to alcohol	1
39	12.0728	$-C_3H_8 + O_2$	Tert-butyl to acid	1
40	14.0157	$-CH_2$	Demethylation	1
41	14.0520	$-C_2H_6 + O$	Ethyl Ether to acid	1
42	15.9772	-S+ O	Thioureas to Ureas	1
43	15.9949	-O	Sulfoxide to Thioether	1
44	17.9661	-Cl + OH	Oxidative Dechlorination	1
45	17.9906	-F + H	Reductive Deflurination	1
46	18.0106	$-H_2O$	Alcohols dehydration	1
47	18.9984	-F	Defluorination	1
48	26.0520	$-C_3H_6 + O$	Isopropyl to alcohol	1
49	27.9943	-CO	Decarbonylation	1
50	28.0313	$-C_2H_4$	Deethylation	1
51	28.0677	$-C_3H_8 + O$	Propyl ether to acid	1
52	29.9742	$-O_2 + H_2$	Nitro reduction	1
53	30.0106	$-CH_2O$	Hydroxymethylene loss	1
54	33.9610	-Cl + H	Reductive Dechlorination	1
55	34.9689	-Cl	Dechlorination	1
56	35.9811	$-F_2 + H_2$	2 Reductive Defluorination	1
57	37.9968	$-F_2$	2 Defluorination	1
58	40.0677	$-C_4H_8 + O$	Tert-butyl to alcohol1	1
59	42.0470	$-C_3H_6$	Isopropyl dealkylation	1
60	44.9851	$-NO_2 + H$	Hydrolysis of Nitrate Esters	1
61	44.9977	-COOH	Decarboxylation	1
62	56.0626	$-C_4H_8$	Tert-butyl to dealkylation	1
63	61.9156	-Br + OH	Oxidative debromination	1
64	67.9220	$-Cl_2 + H_2$	2 Dechlorination + H	1
65	67.9874	$-CF_3 + H$	Loss of Trifluoromethyl	1
66	69.9377	$-Cl_2$	2 Dechlorination	1
67	77.9105	-Br + H	Debromination + H	1
68	78.9183	-Br	Debromination	1
69	90.0470	$-C_7H_6$	Debenzylation	1
70	155.8210	$-Br_2 + H_2$	2 Debromination + H	1
71	157.8367	$-Br_2$	2 Debromination	1

Table S8

-

Supplementary Note 1

```
setwd("D:/同步个人档案/SynologyDrive/实验/原始数据")

#install.packages('readxl')
#install.packages('dplyr')
library(readxl)
library(dplyr)
#install.packages("ggstar")
library(ggstar)
library(ggplot2)

#计算 333 种皂苷虚拟代谢产物的 M.W

df1 <- read_excel('71 种代谢反应.xlsx',sheet = 2) %>% data.frame()
df2 <- read_excel("绞股蓝皂苷及苷元.xlsx",sheet = 3) %>% data.frame() %>% na.omit()

nrow(df2)
head(df1)
```

```r
head(df2)
df1[,1]
df2[,6] <- as.numeric(df2[,6])
str(df1[,1])
str(df2)
da <- ''

i = 1
for (i in 1:nrow(df2)) {
  da1 <- df2[,6][i]+df1[,1][1:31] %>% data.frame()
  da2 <- df2[,6][i]-abs(df1[,1][32:nrow(df1)]) %>% data.frame()
  da3 <- rbind(da1,da2)
  da3 <- data.frame(value = da3,Formula = df2[,3][i],Compounds=df2[,2][i],Mass=df2[,4][i])
  da <- rbind(da,da3)
}
da <- da[-1,]
colnames(da)[1] <- 'M.W'

#保留上面处理的文件
```

```
xx <- data.frame(value = NULL)
str(xx)
#install.packages('openxlsx')
library(openxlsx)
write.xlsx(da,'333 种皂苷及苷元的 71 种预测代谢产物.xlsx')
```

Supplementary Note 2

```
setwd("D:/同步个人档案/SynologyDrive/实验/原始数据")

#install.packages('readxl')
#install.packages('dplyr')
library(readxl)
library(dplyr)
#install.packages("ggstar")
library(ggstar)
library(ggplot2)
```

```
#读取文件

df1 <- read_excel(" 333 种皂苷及苷元的 71 种预测代谢产物.xlsx ", sheet = 1) %>% data.frame() %>% na.omit()
df2 <- read_excel("  绞股蓝皂苷及苷元.xlsx ", sheet = 3) %>% data.frame()

#删除 M.W 重复行
Unique1 <- df1 %>% distinct(M.W, .keep_all = TRUE)
Unique2 <- df2 %>% distinct(M.W, .keep_all = TRUE)
str(Unique1)

a1 <- strsplit(as.character(Unique1[,6]),split = '.',fixed = T)
x1 <- sapply(a1, function(a1) {a1[1]})
x1<-as.numeric(x1)
y0 <- sapply(a1, function(x) {x[2]})
y <- as.numeric(paste0('0','.',y0))
y1 <- y*1000

a2 <- strsplit(as.character(Unique2[,1]),split = '.',fixed = T)
```

```
x2 <- sapply(a2, function(x) {x[1]})
x2<-as.numeric(x2)
y3 <- sapply(a2, function(x) {x[2]})
y4 <- as.numeric(paste0('0','.',y3))
y2 <- y4*1000

df3 <- cbind.data.frame(x1,y1)
df4 <- cbind.data.frame(x2,y2)
df3 <- transform(df3,group="KN")
df4 <- transform(df4,group="UP")
colnames(df4) <- c('x1','y1','group')

ncol(df3)
ncol(df4)
df <- rbind.data.frame(df4,df3)

#改图形字体、字号、颜色等

if (!require("pacman")) install.packages("pacman")
pacman::p_load(dplyr,readxl,ggplot2,mixOmics,data.table,tidyr,Cairo,showtext,ggrepel,ggpubr,pheatmap)
```

```
showtext_auto(enable=T)
font_add("TNM","timesbd.ttf")
font_add('ST','simsun.ttc')
windowsFonts()
windowsFonts(
  KT = windowsFont('楷体'),
  ST = windowsFont('宋体'))


windowsFonts()


themes <-
  theme(panel.background = element_rect(fill = 'white'),
        title = element_text(size=19,family = 'ST',color="black",face = 'italic'),
        axis.text.x = element_text(size=18,family="TNM",color="black"),
        axis.text.y = element_text(size=18,family="TNM",color="black"),
        axis.title.x= element_text(size=20,family="TNM",hjust=0.5),
        axis.title.y= element_text(size=20,family="TNM",hjust=0.5),
        legend.text=element_text(color="black",size=15,family = "TNM"),
```

```r
        legend.title = element_text(color="black", size=18,family = "TNM"),
        plot.title = element_text(hjust = 0.5,face = 'italic'),
        strip.text.x = element_text(size=18, angle=0),
        strip.text.y = element_text(size=18),
        strip.background = element_rect(colour="black", fill="white"),
        #axis.line = element_line(arrow = arrow(length = unit(0.5, 'cm'))))
        axis.line.x=element_line(linetype=1,color="black",linewidth=.4),
        axis.line.y=element_line(linetype=1,color="black",linewidth=.4))


#画多边形图
library(plyr)
find_hull <- function(df) df[chull(df$x1, df$y1),]
hulls <- ddply(df, "group", find_hull)

ggplot()+
  geom_polygon(data = hulls[-(1:11),],aes(x=x1, y=y1),
               #colour='black',size=0.1,alpha = .01) +
               colour="blue",size=0.4,alpha = 0) +
  geom_point(data=df4,
```

```
        aes(x=x1,y=y1),
        size=2,
        shape=18,
        fill="blue",
        #fill="#528FAD",
        color="blue",alpha = 1,stroke=0.000001)+
geom_point(data=df3,
          aes(x=x1,y=y1),
          size=1.7,
          shape=17,
          fill="red",
          #fill="#e76254",
          color='red',alpha = 1,stroke=0.000001)+
scale_x_continuous(breaks = seq(0,2000,200))+
scale_y_continuous(breaks = seq(0,800,100))+
#theme(legend.position = "righttop",
      #legend.text = element_text(family="TNM",face="italic"))+
theme(panel.background = element_rect(fill = 'white'),
      axis.line = element_line(arrow = arrow(length = unit(0.2, 'cm'))),
      axis.text.x = element_text(size=13,family="TNM",color="black"),
```

```
        axis.text.y = element_text(size=13,family="TNM",color="black"),
        axis.title.x= element_text(size=14,family="TNM",hjust=0.5),
        axis.title.y= element_text(size=14,family="TNM",hjust=0.5))+
       #axis.line.x=element_line(linetype=1,color="black",linewidth=.4),
       #axis.line.y=element_line(linetype=1,color="black",linewidth=.4),
       #axis.line = element_line(arrow = arrow(length = unit(0.2, 'cm'))))+
  labs(x="Mass Range (Da)",y="Mass Defect (mDa)")

ggsave(filename = '333 种皂苷及苷元的 71 种预测代谢产物多边形图.pdf',width = 12,height = 9)
ggsave(filename = '333 种皂苷及苷元的 71 种预测代谢产物多边形图.TIFF',width = 12,height = 9)

xx <- data.frame(value = NULL)
str(xx)

install.packages('openxlsx')
library(openxlsx)
write.xlsx(hulls,'hulls_333.xlsx')
```

Supplementary Note 3

```
setwd("D:/同步个人档案/SynologyDrive/实验/实验数据/20230825-NEG-XQ（0-1-24-3）")

library(readxl)
library(dplyr)
library(sp)
library(xlsxjars)
library(openxlsx)
library(rJava)


df0<-read_excel("20230825-NEG-XQ (0-1-24-3)_1.xlsx", sheet=1) %>% data.frame(check.names = F)
nrow(df0)
df0 <- df0[-1,]
name <- colnames(df0)[-1]
length(name)
df1 <- data.frame(na.omit(df0[,c(1,5)]))
nrow(df1)
```

```
colnames(df1)

df <- read_excel('hulls_333.xlsx',sheet = 1) %>% data.frame(check.names = F)
df2<-df[-(1:11),]



i = 1
for (i in 6:ncol(df0)) {
  da <- data.frame(na.omit(df0[,c(1,i)]))
  da1 <- anti_join(da,df1,by = 'Sample.Name')
  a <- strsplit(da1$Sample.Name,split = '/')
  a1 <- sapply(a, function(x) {x[1]})
  a2 <- strsplit(a1,split = '.',fixed = T)
  x <- sapply(a2, function(x) {x[1]}) %>% as.numeric()
  y0 <- sapply(a2, function(x) {x[2]})
  y1 <- paste0('0','.',y0) %>% as.numeric()
  y <- y1*1000
  data <- data.frame(x = x,y = y,Sample.Name = da1$Sample.Name,Intensity = da1[,2])
  data2 <- point.in.polygon(data$x, data$y,df2[,1],df2[,2]) %>% data.frame()
  colnames(data2) <- 'result'
```

```
    result <- cbind(data,data2)
    write.csv(result,file = paste0(colnames(da)[2],'.','csv'))
}
```
